# Exploring Dimethyl Carbonate as a Green and Efficient Solvent for Highly Regioselective Iodination of Arylboronic Acids

**DOI:** 10.1021/acsomega.5c13454

**Published:** 2026-04-10

**Authors:** Alessandro Santarsiere, Michele Loriso, Francesco Ambrosio, Lucia Chiummiento

**Affiliations:** † Department of Basic and Applied Sciences, 19006 University of Basilicata, Via dell’Ateneo lucano 10, 85100 Potenza, Italy; ‡ Department of Health Sciences, 19006 University of Basilicata, Via dell’Ateneo lucano 10, 85100 Potenza, Italy

## Abstract

A mild and efficient method for the regioselective iodination of arylboronic acids in dimethyl carbonate is reported. Dimethyl carbonate, a well-known green solvent, promotes high regioselectivity and excellent yields, performing efficiently on both activated and nonactivated arylboronic acids, regardless of the position and nature of the substituents.

## Graphical abstract

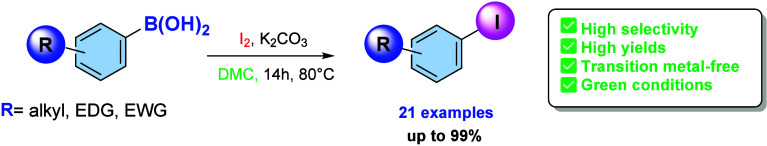

## Introduction

Dimethyl carbonate (DMC) is a well-known green reagent; it is a nonpolar aprotic solvent with good miscibility with water, low toxicity, and rapid atmospheric biodegradability.^1a^ It is considered a potential replacement for solvents such as methyl ethyl ketone, ethyl acetate, methyl isobutyl ketone, and acetonitrile.^1b^ DMC has been increasingly applied in organic synthesis as a viable alternative to chlorine-based reagents,^2^ with numerous green procedures reported for the preparation of pharmaceuticals, polymers, and fragrances;^3^ furthermore, its relatively high dielectric constant makes DMC a suitable electrolyte component in lithium-ion batteries, and it is also being explored as a sustainable alternative to fossil-derived fuels.^4^ Thanks to its high oxygen content (53%), elevated octane number, excellent biodegradability, and low toxicity, DMC is considered a greener fuel additive when compared to the environmentally harmful methyl *tert*-butyl ether (MTBE).^5^

In addition, DMC qualifies as a green solvent that can be directly synthesized from CO_2_, which contributes to the mitigation of the negative environmental effects of this greenhouse gas including global warming. The transformation of CO_2_ into fuels and fine chemicals thus represents a promising approach for addressing both energy and environmental challenges.^6^ In this context, the direct synthesis of DMC from CO_2_ is a particularly attractive route for the environmentally friendly production of this versatile solvent.^4,5a,7^

Aryl iodides are valuable synthetic intermediates, widely used in organic synthesis due to their role in cross-coupling reactions and the generation of free-radical intermediates.^8^ Beyond their synthetic utility, aryl iodides are also present in various natural products and pharmacologically active compounds.^9,10^ A recent approach to their synthesis involves the *ipso*-substitution of arylboronic acids by *N*-iodosuccinimide (NIS).^11^ However, this uncatalyzed method exhibits limitations in substrate scope: arylboronic acids bearing electron-withdrawing groups often lead to poor yields, even under prolonged reaction times. To overcome these drawbacks, various modified protocols have been reported, including base- or phase-transfer-mediated approaches^12^ and copper-catalyzed *ipso*-iodination reactions.^13^ These methods have recently been used to carry out copper-catalyzed *ipso*-radioiodinations.^14^ More recently, DMC has been employed as the reaction medium in gold-catalyzed *ipso*-iodinations of arylboronic acids with NIS;^5^ nevertheless, under catalyst-free conditions, the reported yields remain low, particularly with deactivated substrates or when the position of the boronic acid favors the formation of regioisomeric mixtures. This outcome likely arises from competing electrophilic aromatic substitution/protodeboronation pathways, which become especially pronounced in substrates bearing electron-donating groups at the *ortho* or *meta* position relative to the boronic acid moiety.^15^

## Results and Discussion

Although numerous studies on the *ipso*-iodination of arylboronic acids have been reported in the literature, the use of DMC in this type of reaction represents a further example of the versatility of this green solvent (Scheme 1). Moreover, DMC appears to play a key role in enhancing both the regioselectivity and the yield of the reaction, particularly with deactivated substrates.

**Scheme 1:**
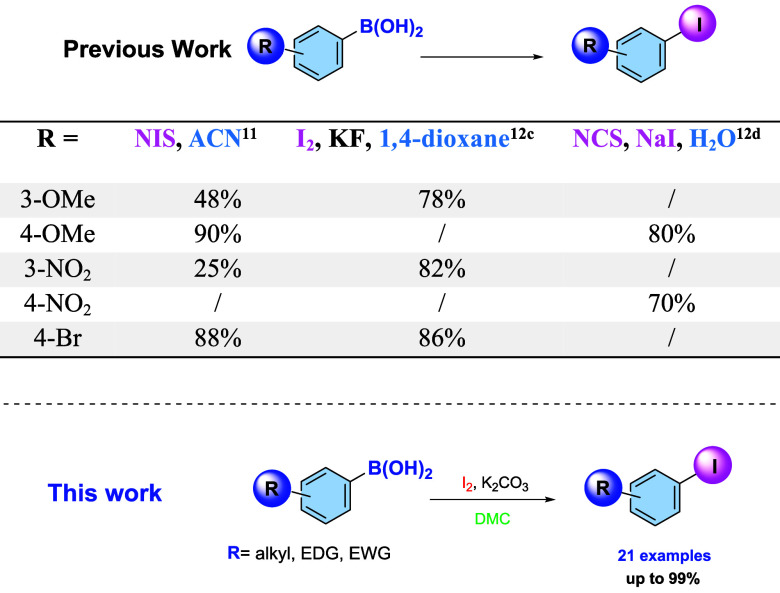
*Ipso*-Iodination of Arylboronic Acids

This work highlights the importance of the involvement of dimethyl carbonate as a solvent. This influence was further determined and confirmed by calculations that corroborate experimental data, supporting the use of DMC as a green alternative to the synthesis of aryl iodides.

3-Methoxyphenylboronic acid (**1a**) was selected as the model substrate to optimize the reaction conditions (see Table 1).

**Table 1:**
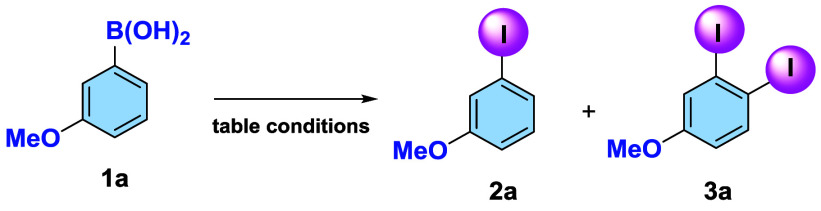

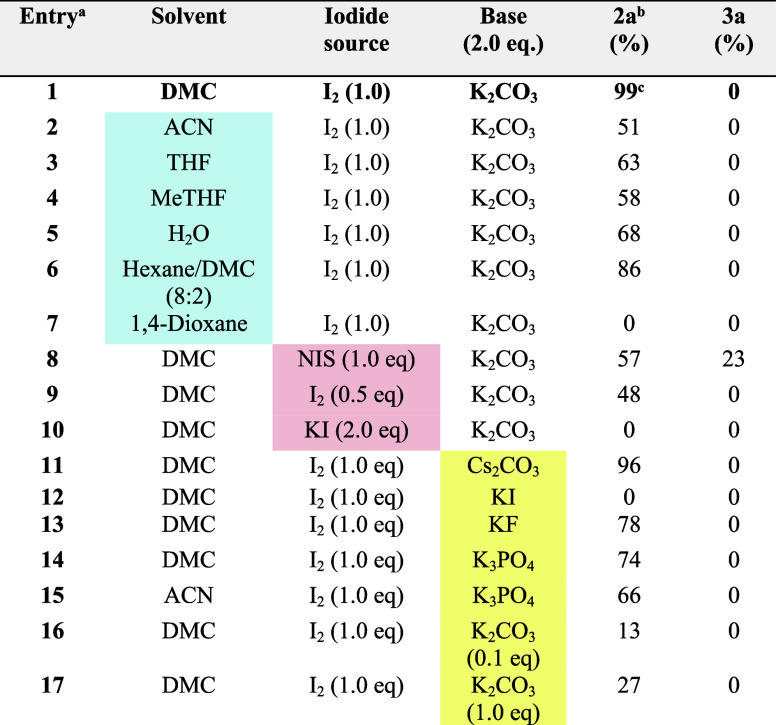
Optimization of Reaction Conditions

a Standard conditions: Substrate (50 mg, 1.0 equiv), I_2_ (1.0 equiv), K_2_CO_3_ (2.0 equiv), solvent (1 mL), 80 °C, 14 h.

b NMR yields.

c Isolated yield.

When ACN was used as the solvent (entry 2), the yield decreased to 51%. Comparable results were obtained with THF, MeTHF, and water (entries 3–5), whereas an 86% yield was achieved in hexane/DMC 8:2 (entry 6).

Finally, the reaction carried out in 1,4-dioxane (entry 7) unexpectedly led to the formation of the corresponding glycol boronic ester without any traces of *ipso*-halogenation.

The use of NIS (entry 8) instead of molecular iodine resulted in both a lower yield and reduced regioselectivity for 3-methoxyphenylboronic acid (**1a**), affording a mixture of **2a:3a** (57:23). The yield further decreased to 48% when 0.1 equiv of I_2_ was employed (entry 9), while no desired product was obtained when KI was used as the iodinating agent (entry 10). Finally, the influence of the base was investigated (entries 11–17): while Cs_2_CO_3_ provided a yield comparable to the standard conditions, the use of KI as the base did not give any reaction product (entry 12), whereas the use of KF (entry 13), acting as a Lewis base which is necessary to activate the phenylboronic acid^12c^ (cf. SI: Energy profiles for the KF-mediated ipso-iodination), afforded a slightly lower yield of 78% compared to K_2_CO_3_ or Cs_2_CO_3_. Finally, the use of K_3_PO_4_ led to a slight decrease in yields (entries 14–15) while the use of 0.1 or 1.0 equiv of K_2_CO_3_ resulted in a significant reduction of the yield.

Regarding the influence of the reaction time on yield, as shown in Scheme 2, the reaction was carried out for 1, 3, 5, 7, and 14 h, resulting in a yield of 52% after 1 h and increasing up to 99% after 14 h.

**Scheme 2:**
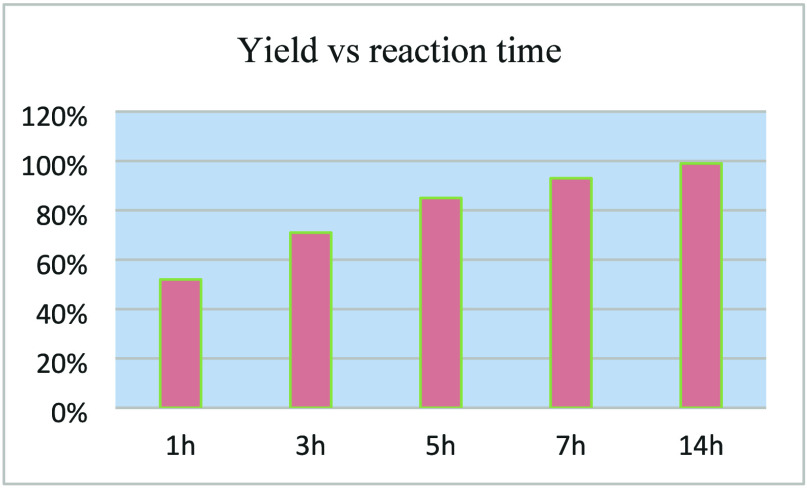
Optimization of Reaction: Yield vs Reaction Time

The I_2_/DMC system appears to have a critical influence on both the yield and regioselectivity. Therefore, the optimized reaction conditions were extended to a variety of substrates (Table 2), affording high yields regardless of the position and nature of the substituent on the substrate.

**Table 2:**
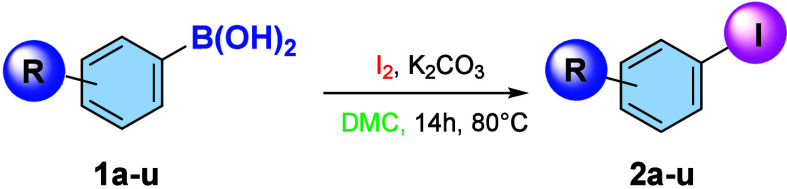

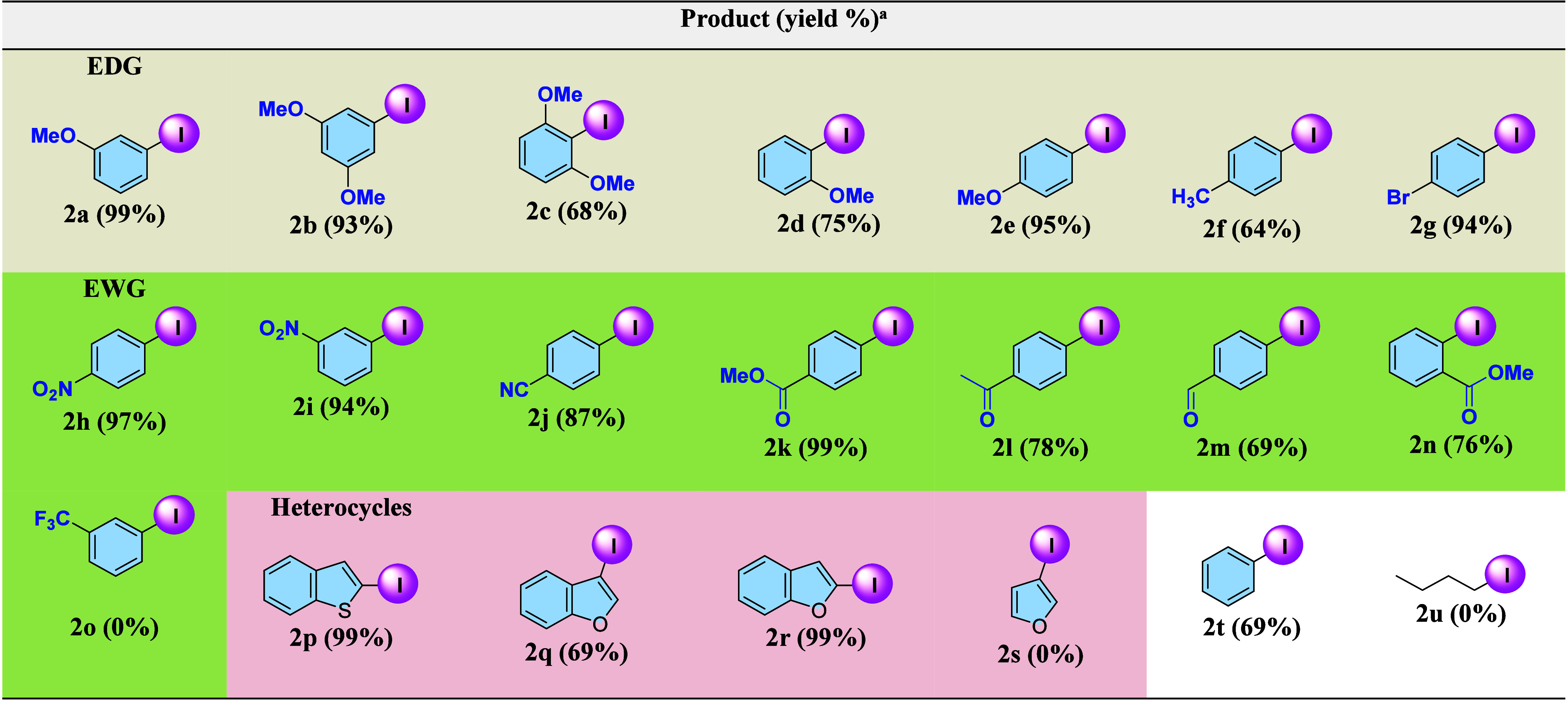
Substrate Scope for Iodination in DMC of Phenylboronic Acids

a Isolated yields.

The iodination of methoxylated substrates (**2a**–**2e**) led to excellent yields regardless of the substituent’s position. Notably, even when the substrate contains two ortho substituents (**2c**), the reaction proceeds with good regioselectivity, despite the *ortho*/*para* position generally being favored over *ortho*/*ortho* iodination. A lower yield was observed for the iodination of *p*-tolyl phenylboronic acid (**2f**), which gave a 64% yield, although no byproducts were detected. Iodination of the *p*-bromo-substituted substrate (**2g**) afforded a high yield of 94%. Interestingly, the reaction also proceeded efficiently with deactivated systems and heterocycles.

The iodination of boronic acids bearing a nitro group (**2h**, **2i**) gave yields of 97% and 94%, respectively. Typically, electron-poor substrates of this type led to very low yields. In particular, the reaction carried out on substrate **1i** using NIS is reported to afford only a 25% yield in ACN^11^ and 16% yield when performed with NIS in DMC in the absence of a gold catalyst.^15^

Good yields were also achieved with substrates bearing *p*-cyano (**2j**), *p*-carboxy ester (**2k**), and *p*-methyl ketone (**2l**) groups, ranging from 78% to 99%. Good yields were also obtained with substrates bearing *p*-formyl (**2m**) and *o*-carboxy ester (**2n**). Finally, iodination of heterocycles afforded an excellent yield with benzothiophene (**2p**) and moderate yield with benzofurans (**2q**–**2r**) or with an unsubstituted system (**2t**).

Unexpectedly, iodination did not afford any product when it was carried out on *m*-CF_3_ (**2o**) and 3-furan (**2s**), which afforded degradation byproducts, and as expected, no product was obtained when the reaction was performed on the aliphatic substrate (**2u)**. Concerning the reaction mechanism, taking into account the obtained results, it could not involve an electrophilic aromatic substitution as in our previous work on *ipso*-formylation of phenylboronic acids.^16^ In order to conceive a viable reaction mechanism (Scheme 3 (a)) and to explain the observed trends for the reaction yield at varying solvents, electronic-structure calculations (cf. SI: Computational Details) were carried out. Again, we focused on *m*-methoxyphenylboronic acid (**1a**) as the reference substrate previously chosen for optimizing the reaction conditions (Table 1).

**Scheme 3:**
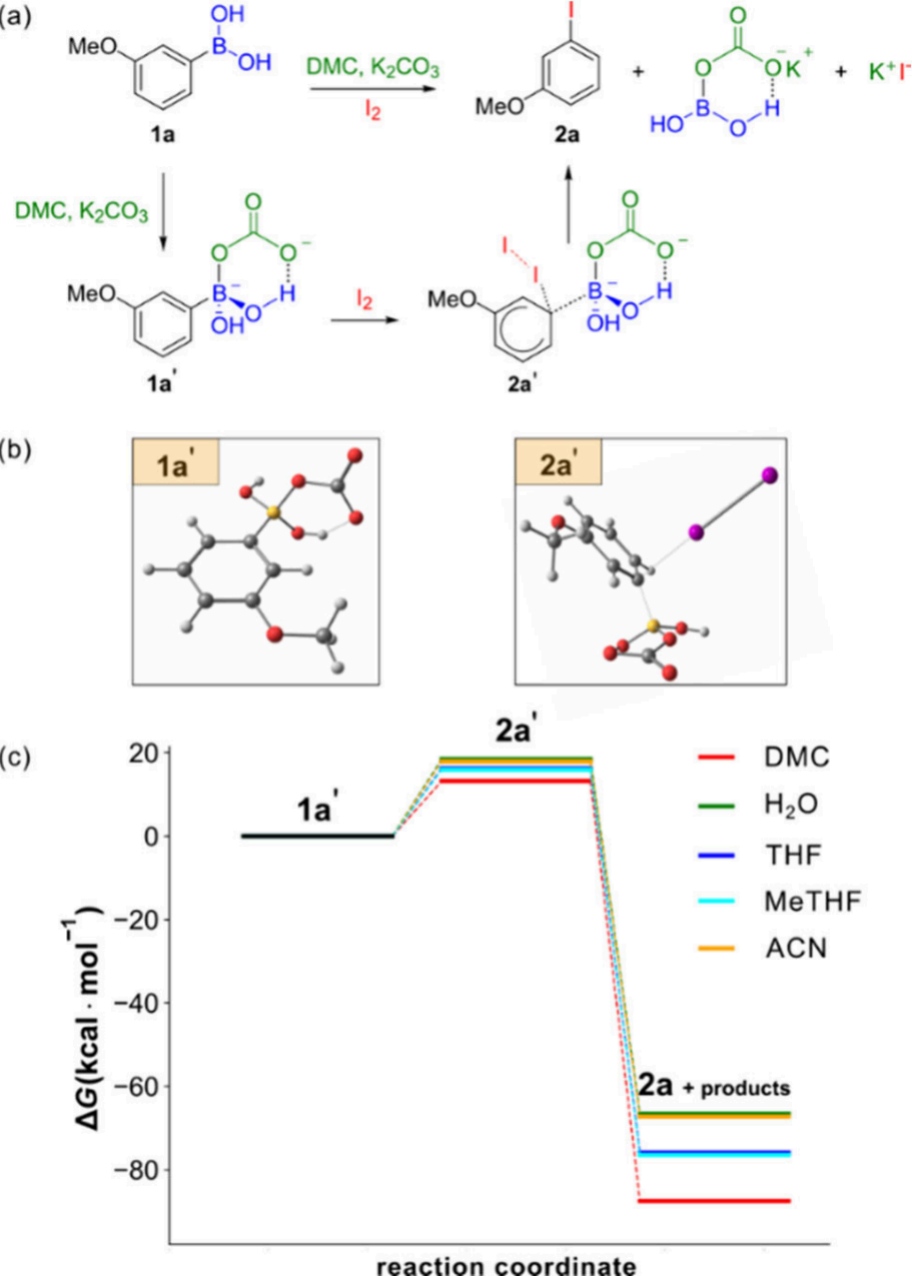
Proposed Mechanism^a^

a a) Proposed reaction mechanism for the iodination of arylboronic acids with I_2_/K_2_CO_3_. b) optimized structures of the intermediate (**1a′**) and the transition state (**2a′**) at the CAM-B3LYP/6–31++G­(d,p) - LANL2DZ level of theory. c) Free energy profiles in different solvents. Free energies referred to intermediate (**1a′**) in each solvent.

We found that initial coordination with the carbonate anion favors the formation of the dianionic intermediate **1a′**. The latter complex features a stabilizing intramolecular hydrogen bond and nucleophilicity toward molecular iodine, thus reflecting the negative charge on the *ipso* carbon (cf. SI: Charge distribution of 1a and 1a′) and the availability of a leaving group on the same site.

The transition state **2a′** features a partial break of C–B and I–I bonds with concomitant C–I bond formation. The mechanism proceeds through the formation of the aryl iodide specimen, potassium iodide and a boron adduct with carbonate.

Our calculations employing an implicit solvation model, revealed that the energetics of the reaction is closely related with the dielectric properties of the solvent.^17,18^ DMC emerged as the most stabilizing medium for both the transition state and the reaction products among the considered solvents, see Scheme 3 (c). Particularly, the transition state in DMC resulted in being more stabilized by at least 5 kcal/mol with respect to the less stabilizing solvent (13.2 vs 18.5 kcal/mol), while products resulted in being much lower in energy (−109 kcal/mol) when compared to other investigated solvents. DMC clearly stands out for its ability to stabilize both the transition state (**2a′**) and the products (**2a**, iodide anion and the carbonate-boron anionic adduct). The proposed reaction mechanism pathway was also verified for *m*-methoxyphenylboronic acid in DMC with KF as a Lewis base (entry 13, Table 1). A similar mechanism was involved in which fluoride gives a coordination to the boron atom, however, without the same stabilization of carbonate, thus justifying the lower reaction yield (cf. SI: Energy profiles for the KF-mediated ipso-iodination).

## Conclusion

The performed calculations revealed how the solvent modulates the reaction energetics and hence the yield and highlighted the activating role of the Lewis base, which operates through a coordinating interaction with key intermediates. Overall, the present analysis corroborates the experimental data, which support the use of DMC as a green alternative for the synthesis of aryl iodides.

In conclusion, this newly developed method for the *ipso-*iodination of arylboronic acids in dimethyl carbonate without a transition-metal catalyst operates efficiently even on nonactivated or activated systems. The use of dimethyl carbonate in this type of reaction represents a further example of its versatility.

## Supplementary Material



## Notes

Experimental procedures, analytical data for all compounds, NMR spectra and computational details (PDF)
