# Supplementary material for: Exploring Dimethyl Carbonate as a Green and Efficient Solvent for Highly Regioselective Iodination of Arylboronic Acids
Source: ACS Omega. 2026 Apr 10;11(15):23166–70. doi: 10.1021/acsomega.5c13454 (PMC13103830; doi:10.1021/acsomega.5c13454)
Supplement: Supplementary file 1 [file ao5c13454_si_001.pdf]

SUPPORTING INFORMATION

Exploring Dimethyl Carbonate as a Green And Efficient Solvent for Highly Regioselective Iodination of Arylboronic Acids

Alessandro Santarsiere,[a,*] Michele Loriso,[a] Francesco Ambrosio[a] and Lucia Chiummiento[b,*]

[a]*Department of Basic and Applied Sciences, University of Basilicata, Via dell'Ateneo lucano 10, 85100 Potenza (Italy)*

[b]*Department of Health Sciences, University of Basilicata, Via dell'Ateneo lucano 10, 85100 Potenza (Italy)*

Table of Contents

1. **General Procedure** **S2**
2. **NMR Spectra** **S6**
3. **Computational Details** **S24**
4. **Charge distribution of 1a and 1a'** **S24**
5. **Thermochemical data** **S24**
6. **Energy profiles for the KF-mediated *ipso*-iodination** **S25**
7. **Energy profiles for the ipso-iodination of phenyl trifluoroboronate without base**........**S25**
8. **Optimized structures** **S26**
9. **References** **S29**

1. General procedures

All reagents were supplied by Sigma-Aldrich, TCI and AlfaAesar companies and were used without further purification unless otherwise stated. All reactions were carried out in oven-dried glassware under an argon atmosphere unless otherwise noted. Flash chromatography was performed using 60–200 mesh silica gel. ^{1}H NMR spectra were recorded on Varian 400 MHz at room temperature with $CDCl_3$ as the solvent unless otherwise noted. Chemical shifts are reported in parts per million relative and referenced internally to the residual solvent resonances: 1H NMR spectra to $CDCl_3$ at δ 7.26. Data for ^{1}H NMR are reported as follows: chemical shift, multiplicity (s = singlet, d = doublet, t = triplet, q = quartet, m = multiplet), coupling constants (in Hertz) and integration.

General procedure for *ipso*-iodination: To a mixture of the arylboronic acid (50 mg, 1.0 equiv), potassium carbonate (2.0 equiv), and iodine (1.0 equiv) was added dimethyl carbonate (DMC, 0.3 M). The reaction mixture was stirred at 80 °C for 14 h. After completion, the reaction was cooled to room temperature and diluted with diethyl ether (2.0 mL), then washed with a saturated aqueous solution of potassium thiosulfate to remove excess iodine. The organic layer was dried over anhydrous sodium sulfate, filtered, and concentrated under reduced pressure. The crude residue was purified by filtration through a pad of silica gel and elution with hexane/ethyl acetate (8:2) to afford the desired iodinated product.

1-iodo-3-methoxybenzene (2a)

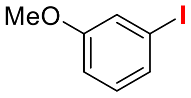

2a

Compound **2a** was obtained using the general procedure for *ipso*-iodination affording **2a** (yellow solid, 74 mg, 99%). ^{1}H NMR (400 MHz, $CDCl_3$) δ = 7.29 (d, J = 7.72 Hz, 1H), 7.01 (t, J = 8.00 Hz, 1H), 6.87 (d, J = 8.04 Hz, 1H), 3.79 (s, 3H) ppm. ^{13}C NMR (100 MHz, $CDCl_3$) δ= 159.91, 130.57, 129.61, 122.78, 113.56, 94.19, 55.18 ppm.

1-iodo-3,5-dimethoxybenzene (2b)

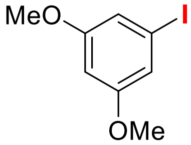

2b

Compound **2b** was obtained using the general procedure for *ipso*-iodination affording **2b** (white solid, 68 mg, 93%). ^{1}H NMR (400 MHz, $CDCl_3$) δ = 6.85 (s, 2H), 6.39 (s,1H), 3.76 (s, 6H) ppm. ^{13}C NMR (100 MHz, $CDCl_3$) δ= 161.49, 116.19, 101.13, 94.54, 55.98 ppm.

2-iodo-1,3-dimethoxybenzene (2c)

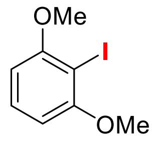

2c

Compound **2c** was obtained from arylboronic acid **1c** (28mg, 0.15 mmol) using the general procedure for *ipso*-iodination and it was purified by column chromatography on silica gel (petroleum ether/AcOEt 8:2) affording **2c** (28 mg, 68%). ^{1}H NMR (400 MHz, $CDCl_3$) δ = 7.24 (t, *J* = 8.00 Hz, 1H), 6.49 (d, *J* = 8.00 Hz, 2H), 3.87 (s, 3H) ppm. ^{13}C NMR (100 MHz, $CDCl_3$) δ= 159.51, 139.15, 129.81, 104.06, 56.55 ppm.

1-iodo-2-methoxybenzene (2d)

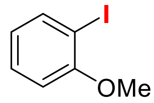

2d

Compound **2d** was obtained using the general procedure for *ipso*-iodination affording **2d** (58 mg, 75%). ^{1}H NMR (400 MHz, $CDCl_3$) δ = 7.78 (dd, *J* = 7.76 Hz, *J* = 1.68 Hz, 1H), 7.31 (dt, *J* = 8.16 Hz, *J* = 1.64 Hz, 1H), 6.83 (d, *J* = 8.28 Hz, 1H), 3.88 (s, 3H) ppm. ^{13}C NMR (100 MHz, $CDCl_3$) δ= 158.14, 139.59, 129.65, 122.61, 111.08, 86.08, 56.39 ppm.

1-iodo-4-methoxybenzene (2e)

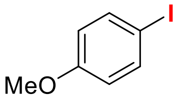

2e

Compound **2e** was obtained using the general procedure for *ipso*-iodination affording **2e** (white solid, 77 mg, 95 %). ^{1}H NMR (400 MHz, $CDCl_3$) δ = 7.55 (d, *J* = 8.8 Hz, 2H), 6.68 (d, *J* = 8.8 Hz, 2H), 3.78 (s, 3H) ppm. ^{13}C NMR (100 MHz, $CDCl_3$) δ= 159.42, 138.17, 116.34, 82.71, 55.31 ppm.

1-iodo-4-methylbenzene (2f)

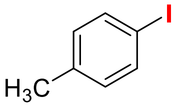

2f

Compound **2f** was obtained from arylboronic acid **1f** (30mg, 0.22 mmol) using the general procedure for *ipso*-iodination and it was purified by column chromatography on silica gel (petroleum ether/AcOEt 8:2) affording **2f** (31 mg, 64 %). ^{1}H NMR (400 MHz, $CDCl_3$) δ = 7.56 (d, *J* = 7.71 Hz, 2H), 6.92 (d, *J* = 7.72 Hz, 2H), 2.29 (s, 3H) ppm. ^{13}C NMR (100 MHz, $CDCl_3$) δ= 137.61, 137.36, 131.34, 90.33, 21.17 ppm.

1-bromo-4-iodobenzene (2g)

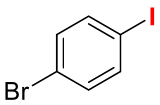

2g

Compound **2g** was obtained using the general procedure for *ipso*-iodination affording **2g** (66 mg, 94 %) ^{1}H NMR (400 MHz, $CDCl_3$) δ = 7.54 (d, *J* = 8.88 Hz, 2H), 7.23 (d, *J* = 8.89 Hz, 2H) ppm. ^{13}C NMR (100 MHz, $CDCl_3$) δ= 139.19, 133.57, 122.33, 92.19 ppm.

1-iodo-4-nitrobenzene (2h)

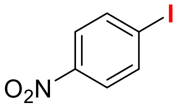

2h

Compound **2h** was obtained using the general procedure for *ipso*-iodination affording **2h** (white solid, 70 mg, 94 %). ^{1}H NMR (400 MHz, $CDCl_3$) δ = 7.95 (m, 4H) ppm. ^{13}C NMR (100 MHz, $CDCl_3$) δ= 147.70, 138.78, 124.94, 102.87 ppm.

1-iodo-3-nitrobenzene (2i)

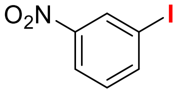

2i

Compound **2i** was obtained using the general procedure for *ipso*-iodination affording **2i** (yellow oil, 72 mg, 97%). ^{1}H NMR (400 MHz, $CDCl_3$) δ = 8.56 (s, 1H), 8.20 (d, *J* = 8.12 Hz, 1H), 8.02 (d, *J* = 7.80 Hz, 1H), 7.29 (t, *J* = 7.80 Hz, 1H) ppm. ^{13}C NMR (100 MHz, $CDCl_3$) δ= 148.92, 143.91, 132.85, 131.15, 123.19, 93.93 ppm.

4-iodobenzonitrile (2j)

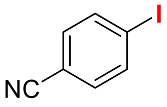

2j

Compound **2j** was obtained using the general procedure for *ipso*-iodination affording **2j** (68 mg, 87 %). ^{1}H NMR (400 MHz, $CDCl_3$) δ = 7.84 (d, *J* = 8.00 Hz, 2H), 7.36 (d, *J* = 8.00 Hz, 2H) ppm. ^{13}C NMR (100 MHz, $CDCl_3$) δ= 138.91, 133.58, 118.65, 112.12, 100.76 ppm.

Methyl 4-iodobenzoate (2k)

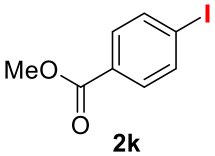

2k

Compound **2k** was obtained using the general procedure for *ipso*-iodination affording **2k** (white solid, 72.5 mg, 99%) ^{1}H NMR (400 MHz, $CDCl_3$) δ = 7.79 (d, *J* = 8.39 Hz, 2H), 7.73 (d, *J* = 8.36 Hz, 2H), 3.90 (s, 3H) ppm. ^{13}C NMR (100 MHz, $CDCl_3$) δ= 167.01, 138.14, 131.45, 130.00, 101.19, 52.75 ppm.

1-(4-iodophenyl)ethan-1-one (2l)

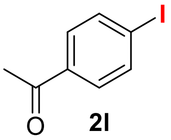

2l

Compound **2l** was obtained using the general procedure for *ipso*-iodination affording **2l** (white solid, 59 mg, 78%). ^{1}H NMR (400 MHz, $CDCl_3$) δ = 7.81 (d, *J* = 8.16 Hz, 2H), 7.64 (d, *J* = 8.16 Hz, 2H), 2.56 (s, 3H) ppm. ^{13}C NMR (100 MHz, $CDCl_3$) δ= 197.44, 137.98, 136.39, 129.82, 101.24, 26.61 ppm.

4-iodobenzaldehyde (2m)

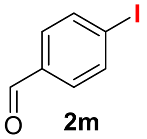

2m

Compound **2m** was obtained using the general procedure for *ipso*-iodination affording **2m** (white solid, 53.1 mg, 69 %). ^{1}H NMR (400 MHz, $CDCl_3$) δ = 9.95 (s, 1H), 7.90 (d, *J* = 8.26 Hz, 2H), 7.58 (d, *J* = 8.03 Hz, 2H) ppm. ^{13}C NMR (100 MHz, $CDCl_3$) δ= 191.58, 138.52, 135.64, 130.93, 102.99 ppm.

Methyl 2-iodobenzoate (2n)

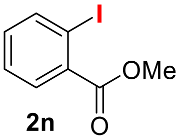

2n

Compound **2n** was obtained using the general procedure for *ipso*-iodination affording **2n** (colorless oil, 55 mg, 76 %). ^{1}H NMR (400 MHz, $CDCl_3$) δ = 7.99 (d, *J* = 7.83 Hz, 1H), 7.80 (d, *J* = 7.86 Hz, 2H), 7.40 (t, *J* = 7.52 Hz, 1H), 7.15 (t, *J* = 7.61 Hz, 1H), 3.93 (s, 3H) ppm. ^{13}C NMR (100 MHz, $CDCl_3$) δ= 167.44, 141.77, 135.48, 133.12, 131.40, 128.35, 94.55, 52.95 ppm.

2-iodobenzo[b]thiophene (2p)

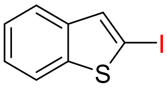

2p

Compound **2p** was obtained using the general procedure for *ipso*-iodination affording **2p** (white solid, 73 mg, 99 %). ^{1}H NMR (400 MHz, $CDCl_3$) δ = 7.77 (d, *J* = 7.23 Hz, 1H), 7.72 (d, *J* = 7.30 Hz, 1H), 7.54 (s, 1H), 7.33-7.27 (m, 2H) ppm. ^{13}C NMR (100 MHz, $CDCl_3$) δ= 144.42, 140.84, 133.84, 124.56, 124.43, 122.32, 121.29, 78.46 ppm.

3-iodobenzofuran (2q)

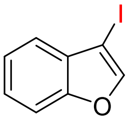

2q

Compound **2q** was obtained using the general procedure for *ipso*-iodination affording **2q** (yellow oil, 52 mg, 69 %). ^{1}H NMR (400 MHz, $CDCl_3$) δ = 7.66 (s, 1H), 7.50 (d, *J* = 8.14 Hz, 1H), 7.44 (d, *J* = 7.42 Hz, 1H), 7.39-7.31 (m, 2H) ppm. ^{13}C NMR (100 MHz, $CDCl_3$) δ= 154.50, 147.13, 129.74, 125.58, 123.61, 121.48, 111.65, 64.40 ppm.

2-iodobenzofuran (2r)

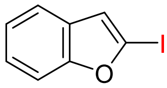

2r

Compound **2r** was obtained using the general procedure for *ipso*-iodination affording **2r** (yellow oil, 75 mg, 99%). ^{1}H NMR (400 MHz, $CDCl_3$) δ = 7.53-7.48 (m, 2H), 7.25-7.20 (m, 2H), 6.96 (s, 1H) ppm. ^{13}C NMR (100 MHz, $CDCl_3$) δ= 158.28, 129.29, 124.35, 123.24, 119.80, 117.33, 110.94, 96.02 ppm.

Iodobenzene (2t)

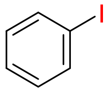

2t

Compound **2t** was obtained using the general procedure for *ipso*-iodination affording **2t** (colorless oil, 58 mg, 69 %) ^{1}H NMR (400 MHz, $CDCl_3$) δ = 7.71 (d, *J*=7.67 Hz, 2H), 7.34 (t, *J*=7.70 Hz, 1H), 7.11 (d, *J*=7.68 Hz, 2H) ppm. ^{13}C NMR (100 MHz, $CDCl_3$) δ= 137.59, 130.38, 127.60, 94.53 ppm

2. NMR Spectra

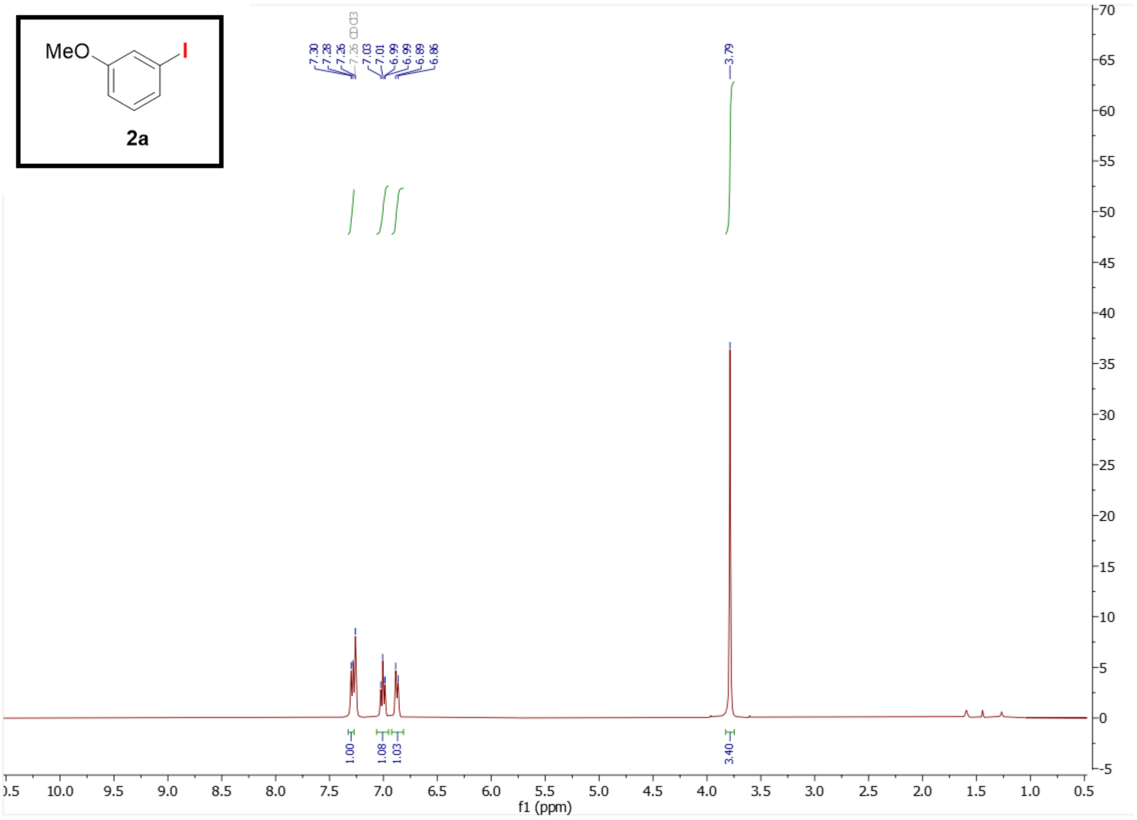

Figure S1. ^{1}H NMR (400 MHz, $CDCl_3$) of **2a**

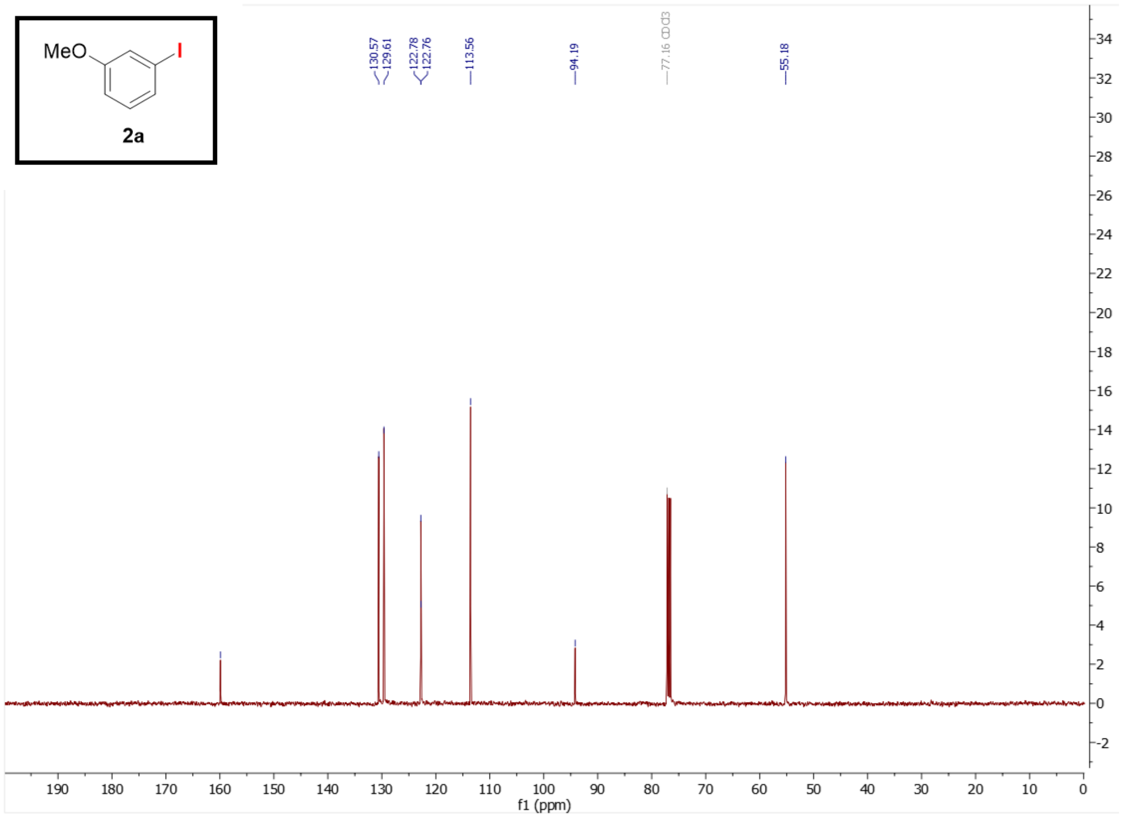

Figure S2. ^{13}C NMR (100 MHz, $CDCl_3$) of **2a**

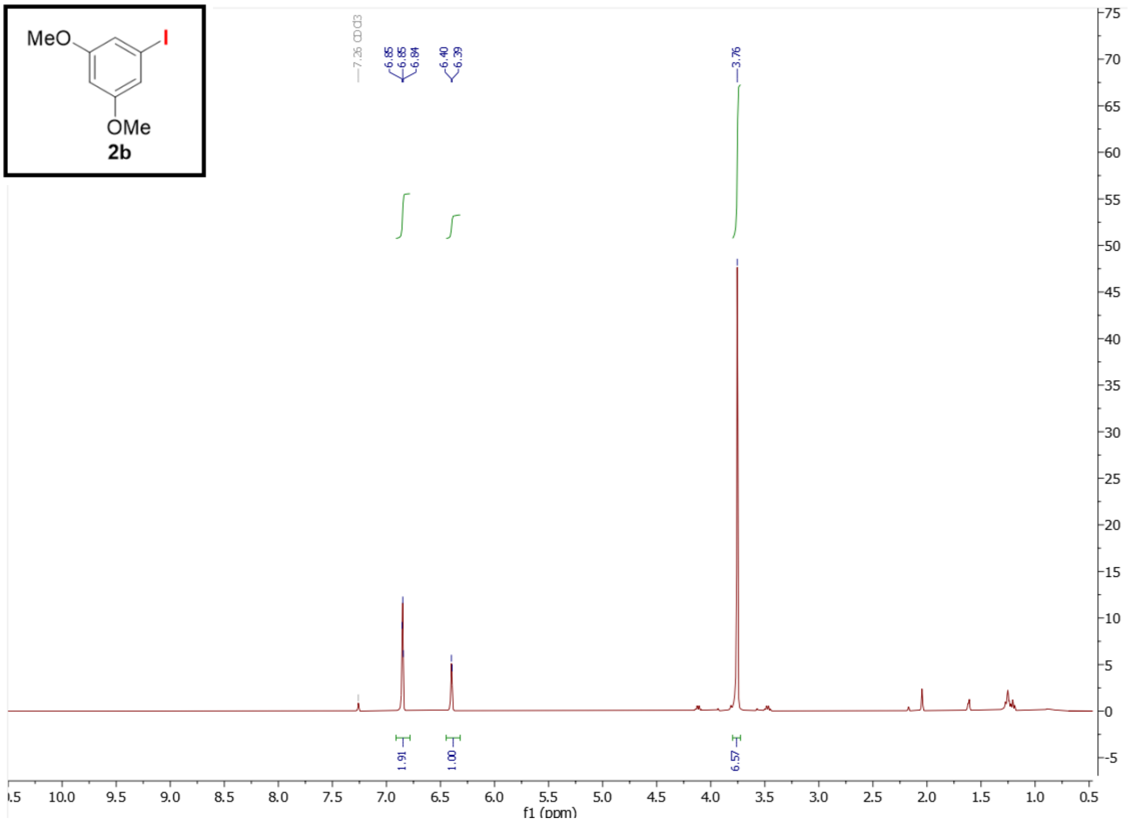

Figure S3. ^{1}H NMR (400 MHz, $CDCl_3$) of **2b**

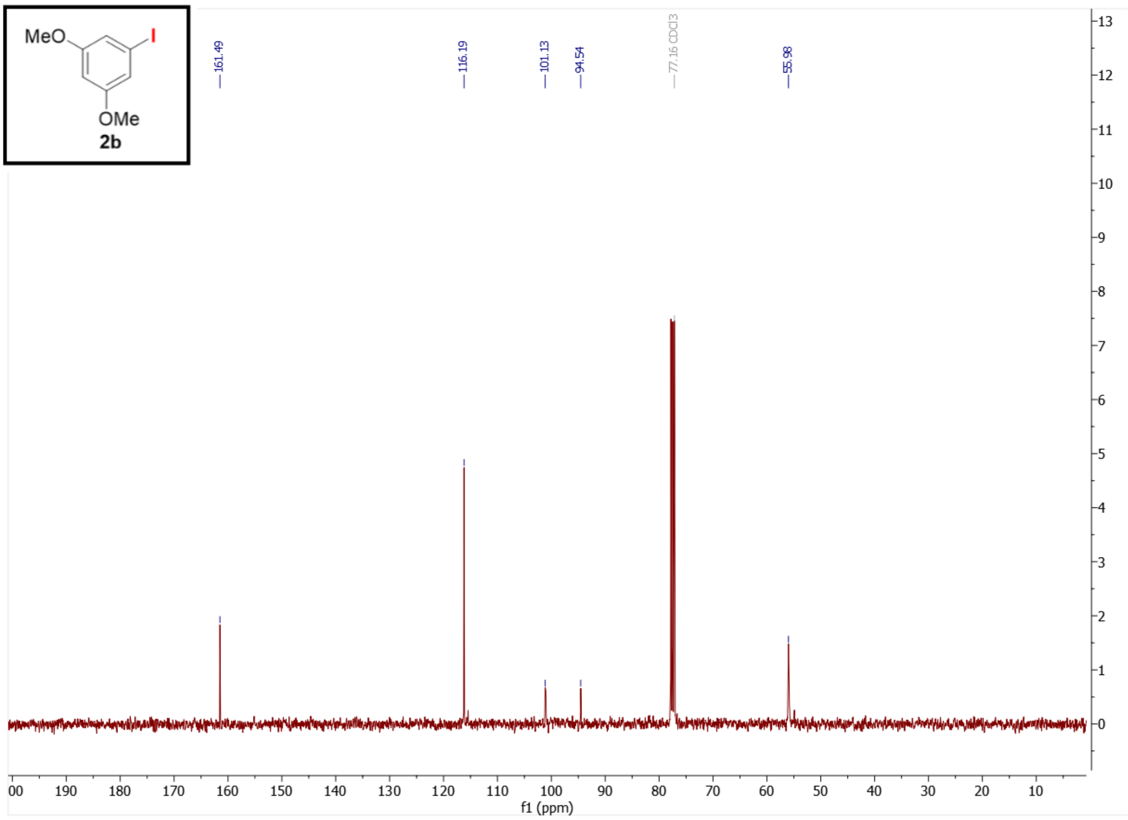

Figure S4. ^{13}C NMR (100 MHz, $CDCl_3$) of **2b**

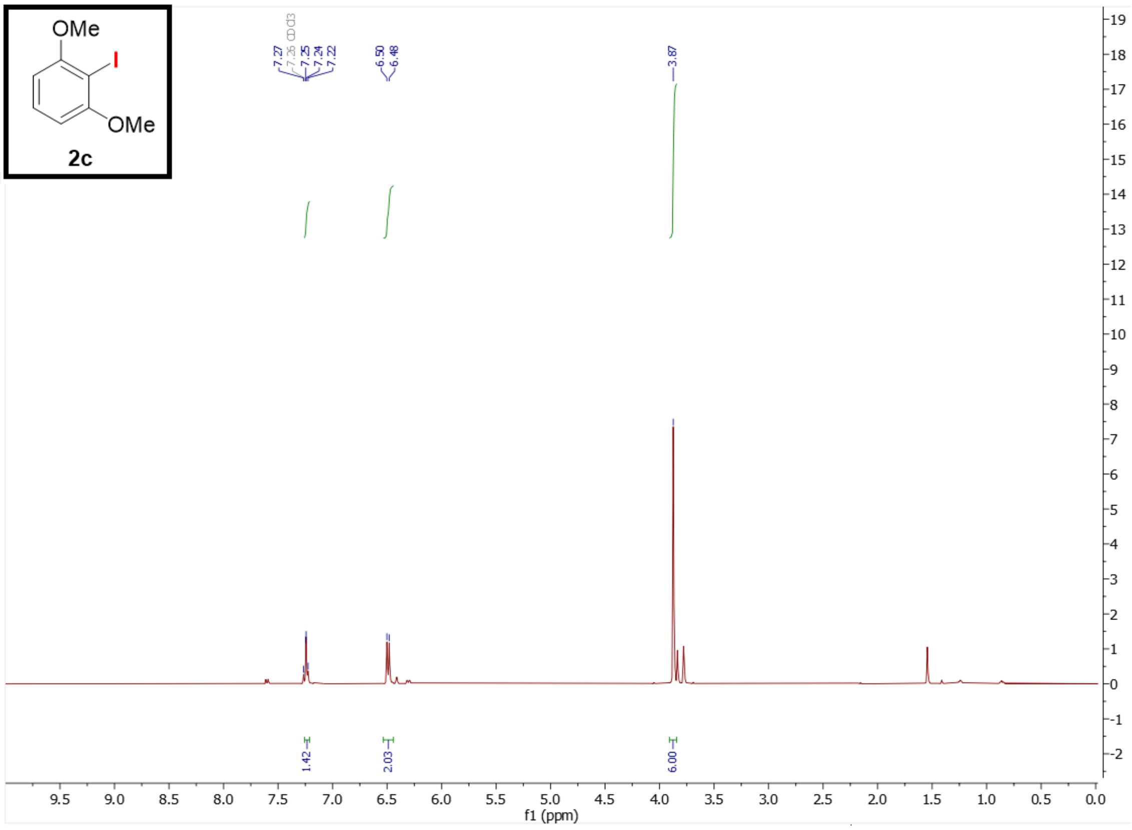

Figure S5. ^{1}H NMR (400 MHz, $CDCl_3$) of **2c**

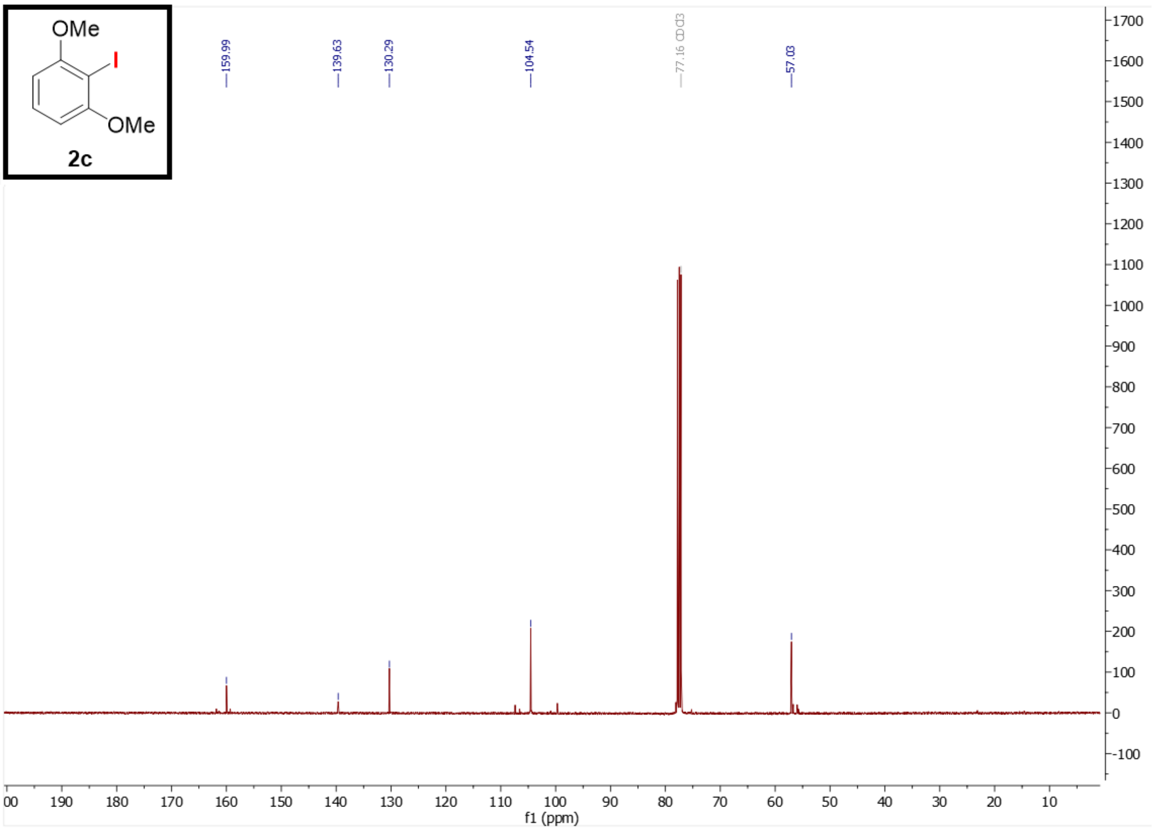

Figure S6. ^{13}C NMR (100 MHz, $CDCl_3$) of **2b**

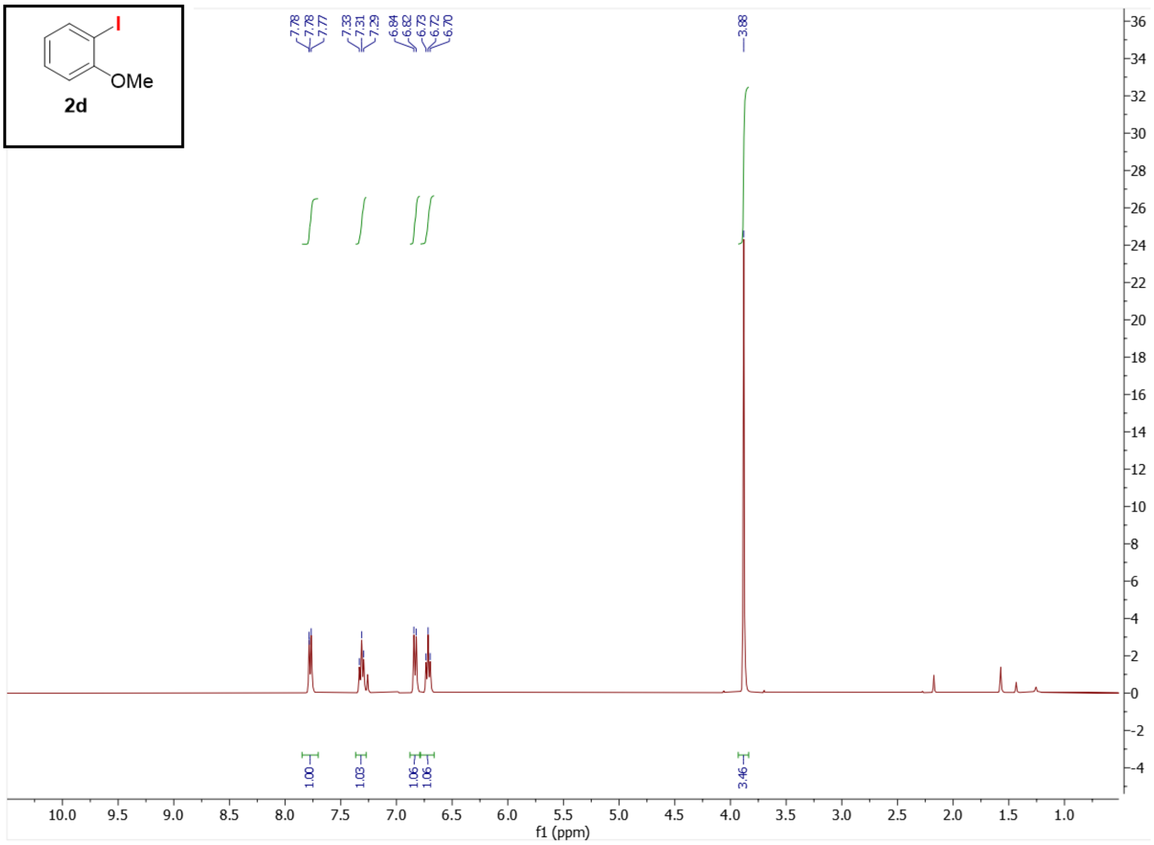

Figure S7. ^{1}H NMR (400 MHz, $CDCl_3$) of **2d**

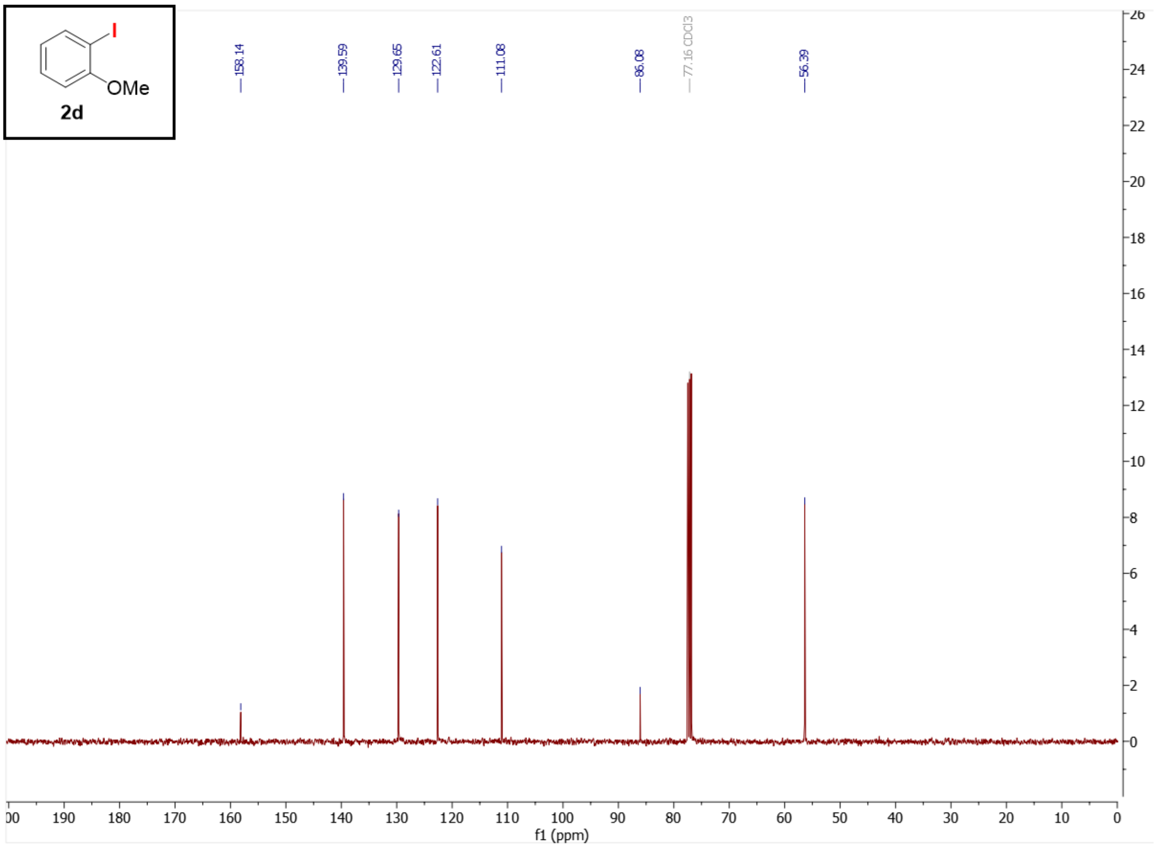

Figure S8. ^{13}C NMR (100 MHz, $CDCl_3$) of **2d**

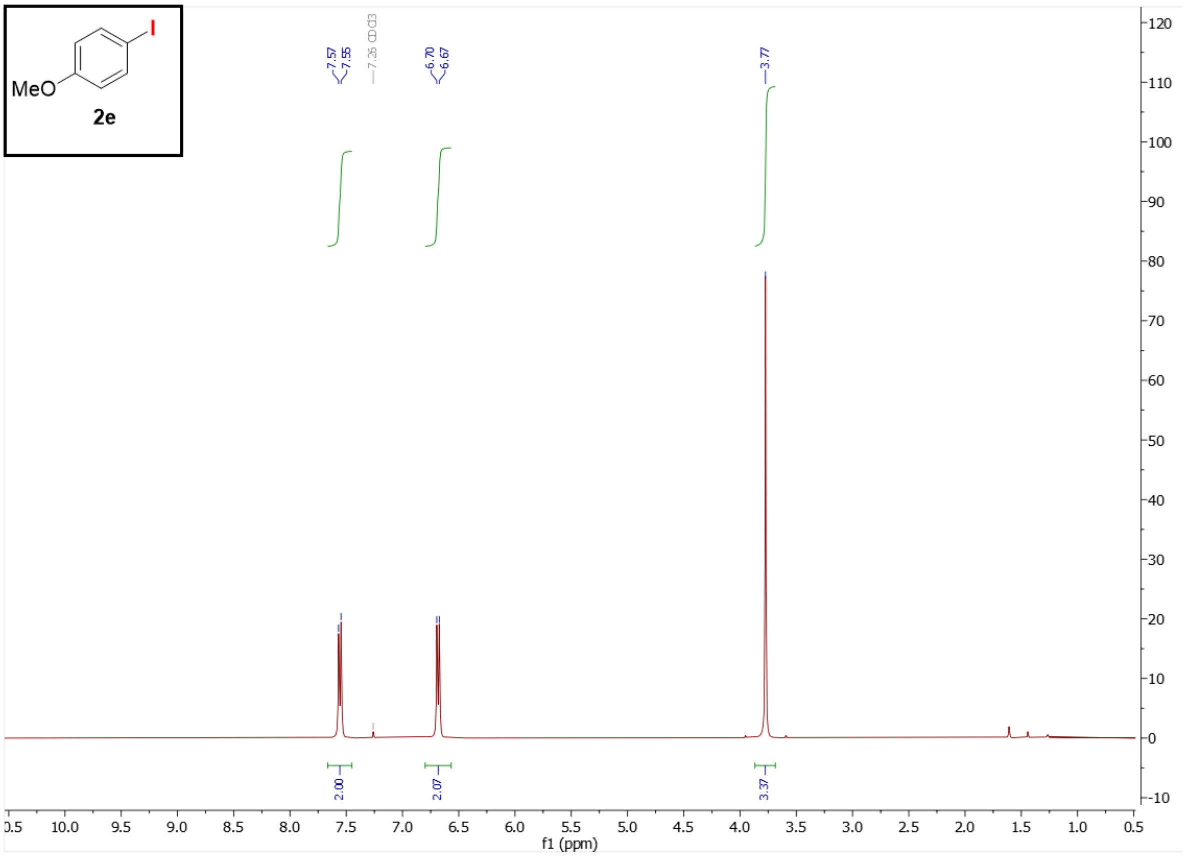

Figure S9. ^{1}H NMR (400 MHz, $CDCl_3$) of **2e**

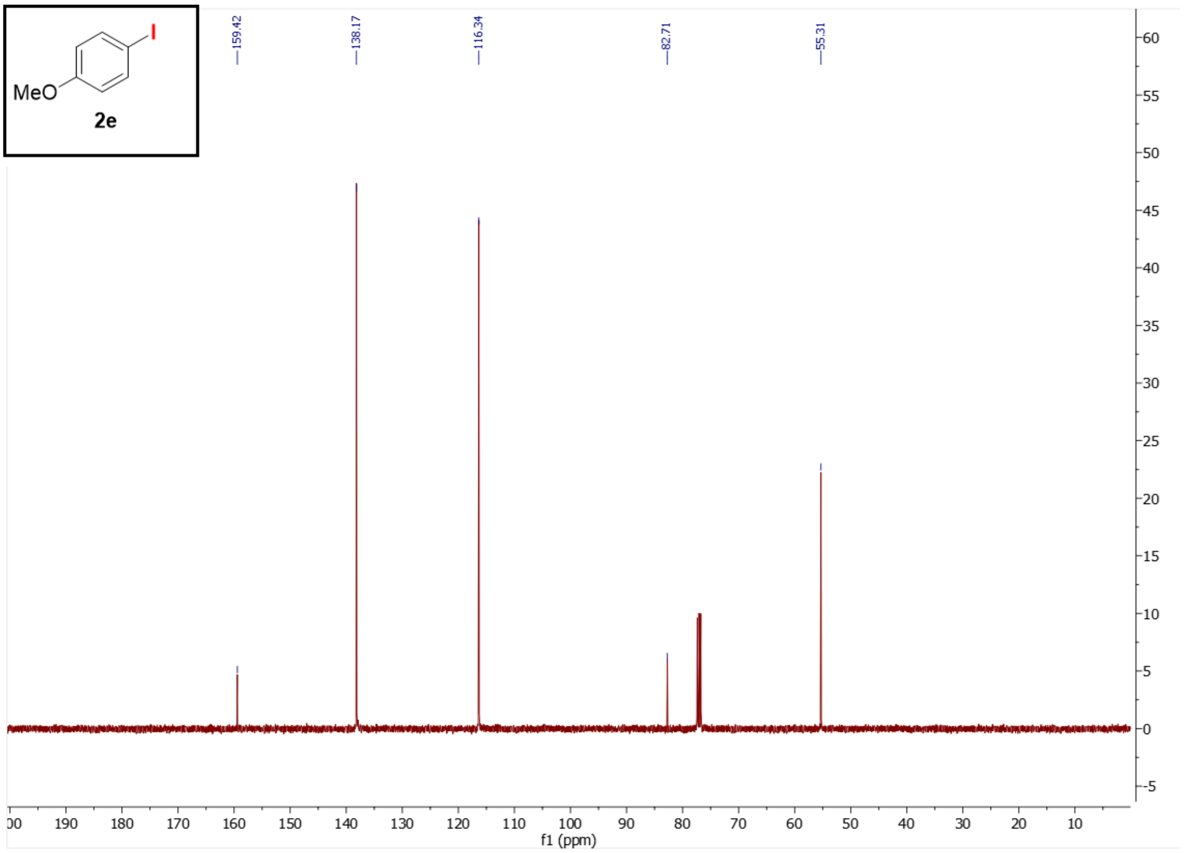

Figure S10. ^{13}C NMR (100 MHz, $CDCl_3$) of **2e**

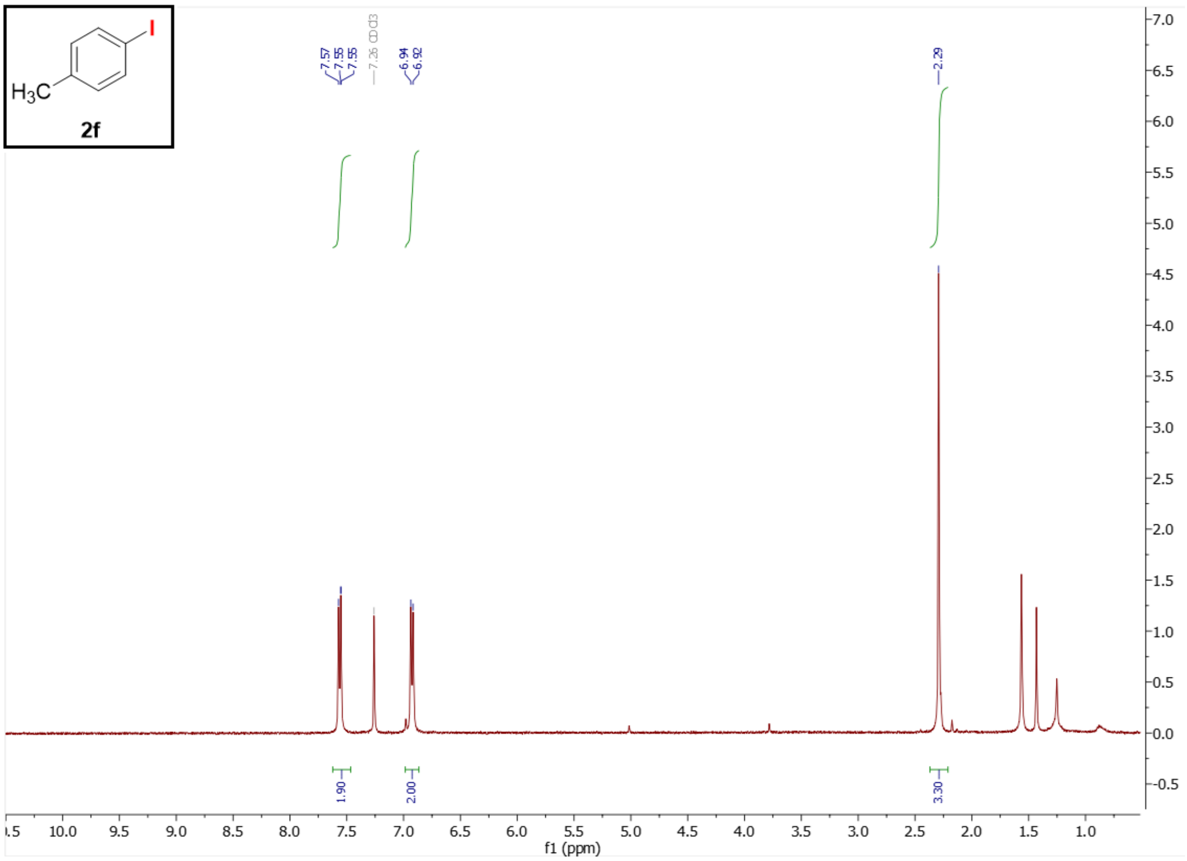

Figure S11. ^{1}H NMR (400 MHz, $CDCl_3$) of **2f**

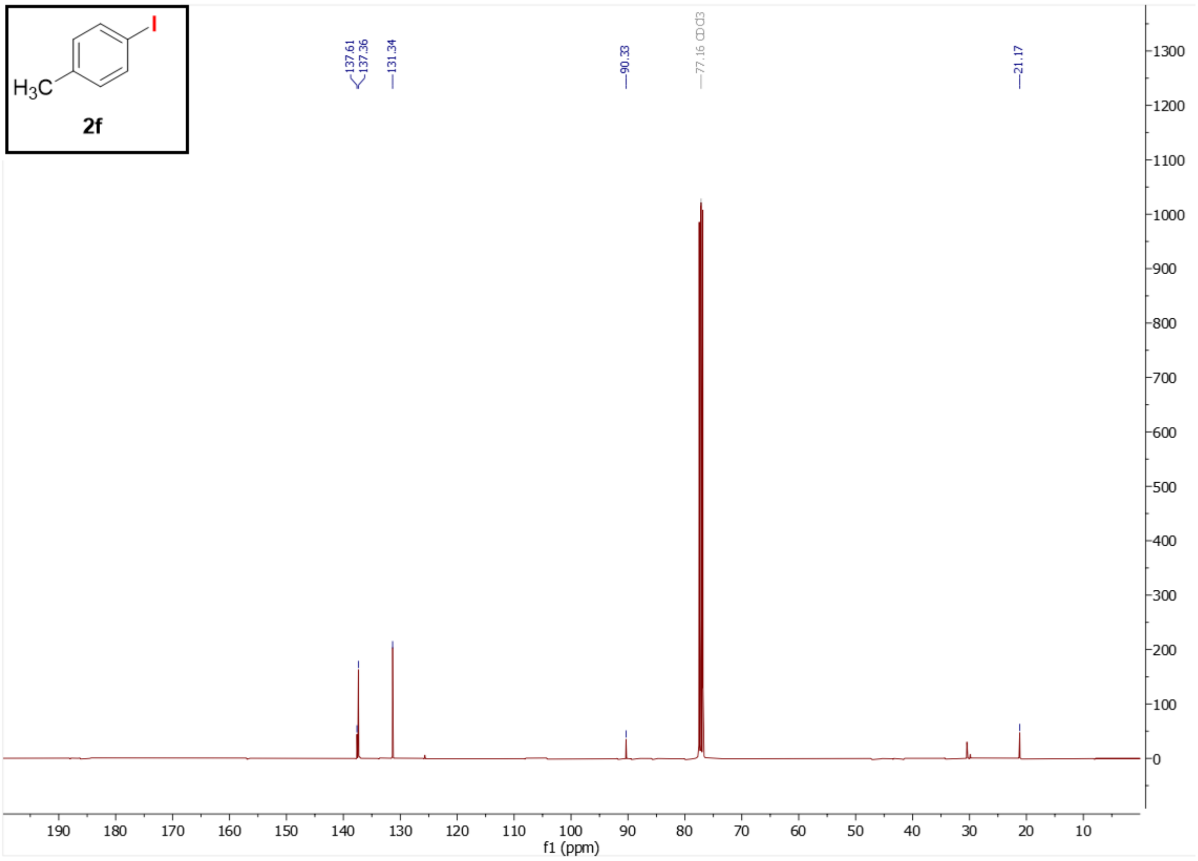

Figure S12. ^{13}C NMR (100 MHz, $CDCl_3$) of **2f**

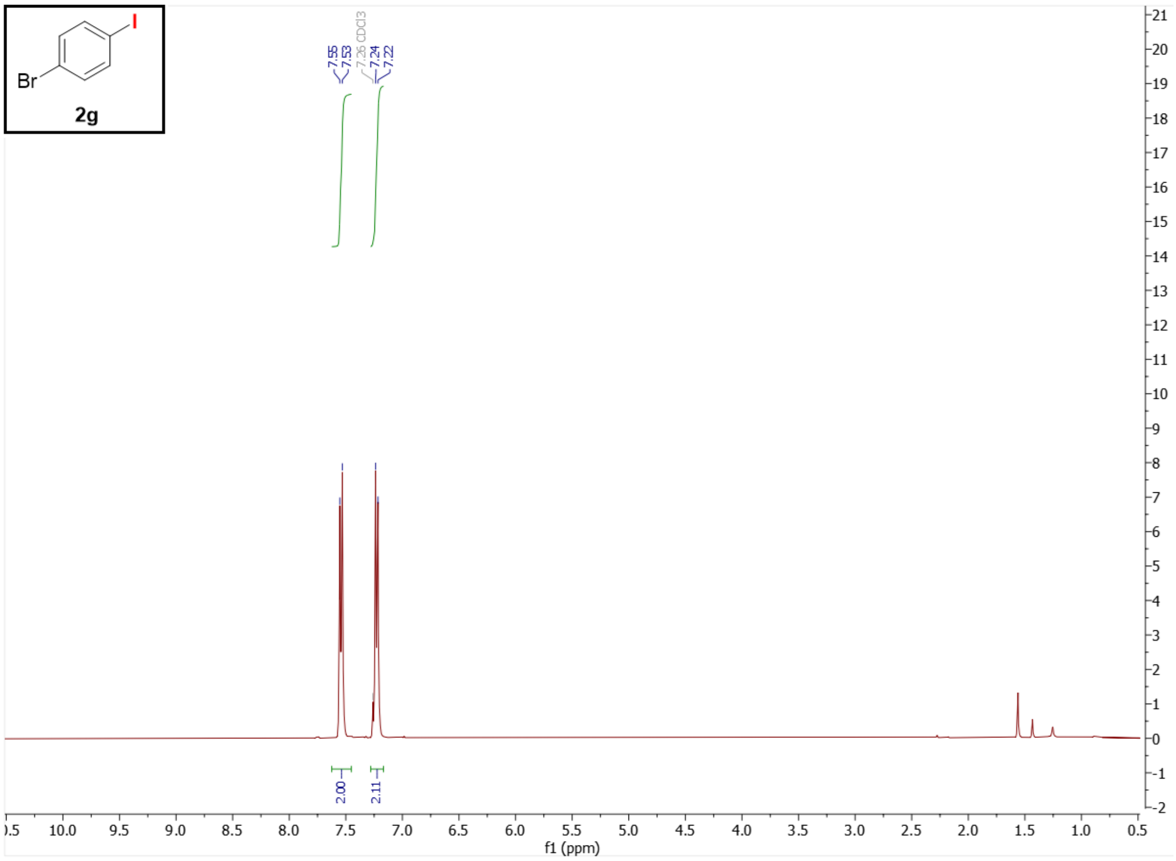

Figure S13. ^{1}H NMR (400 MHz, $CDCl_3$) of **2g**

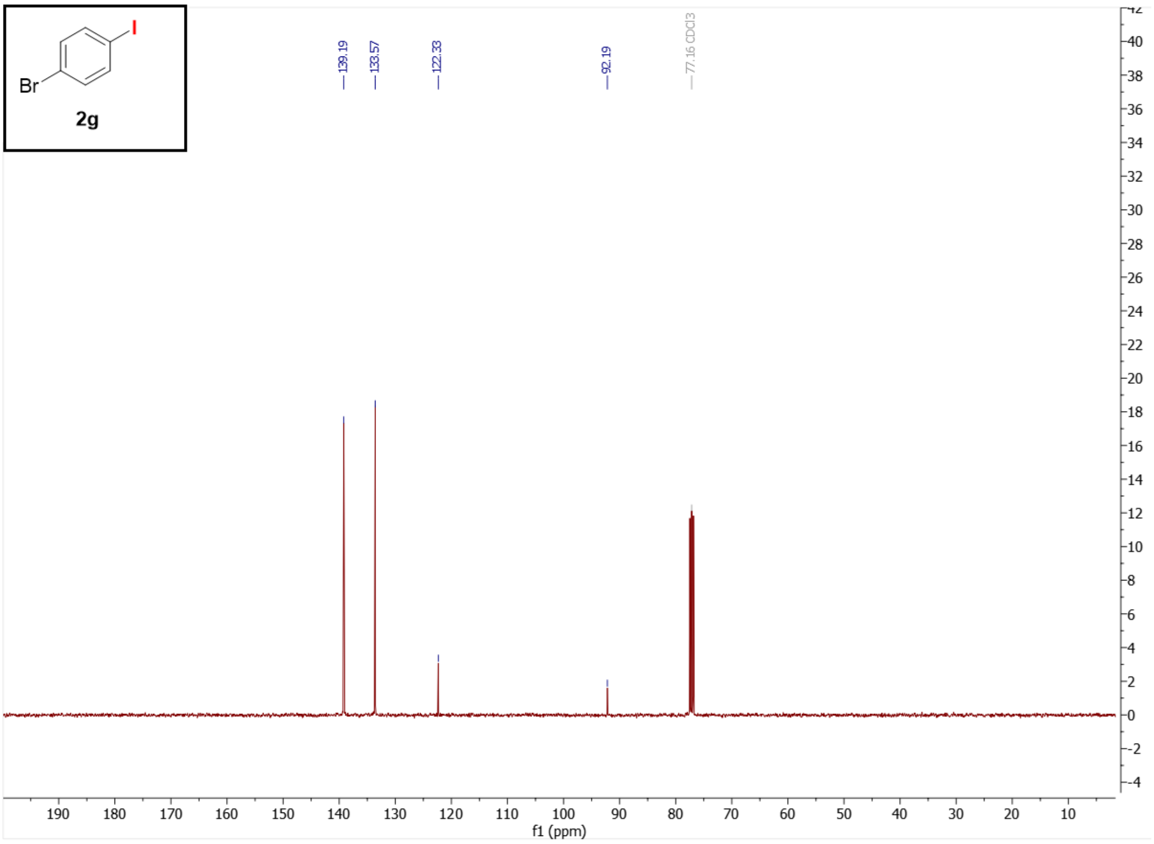

Figure S14. ^{13}C NMR (100 MHz, $CDCl_3$) of **2g**

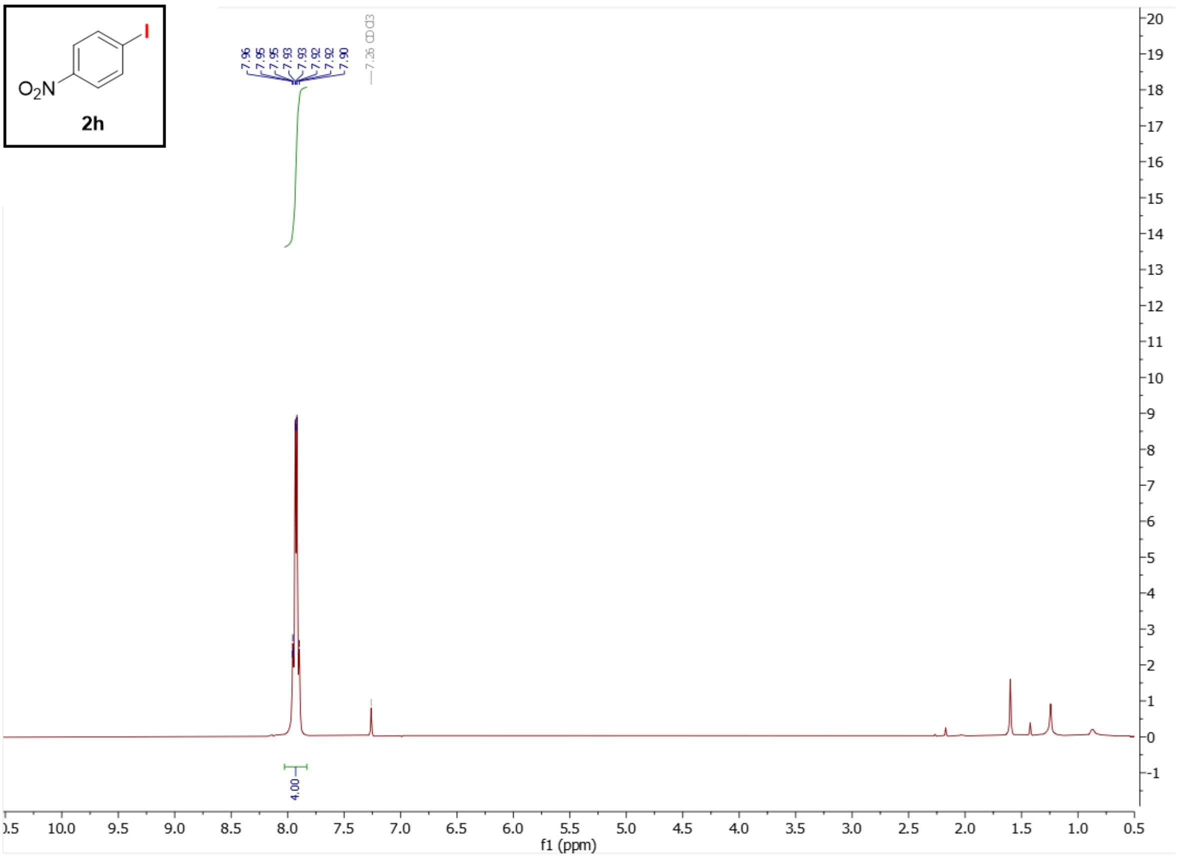

Figure S15. ^{1}H NMR (400 MHz, $CDCl_3$) of **2h**

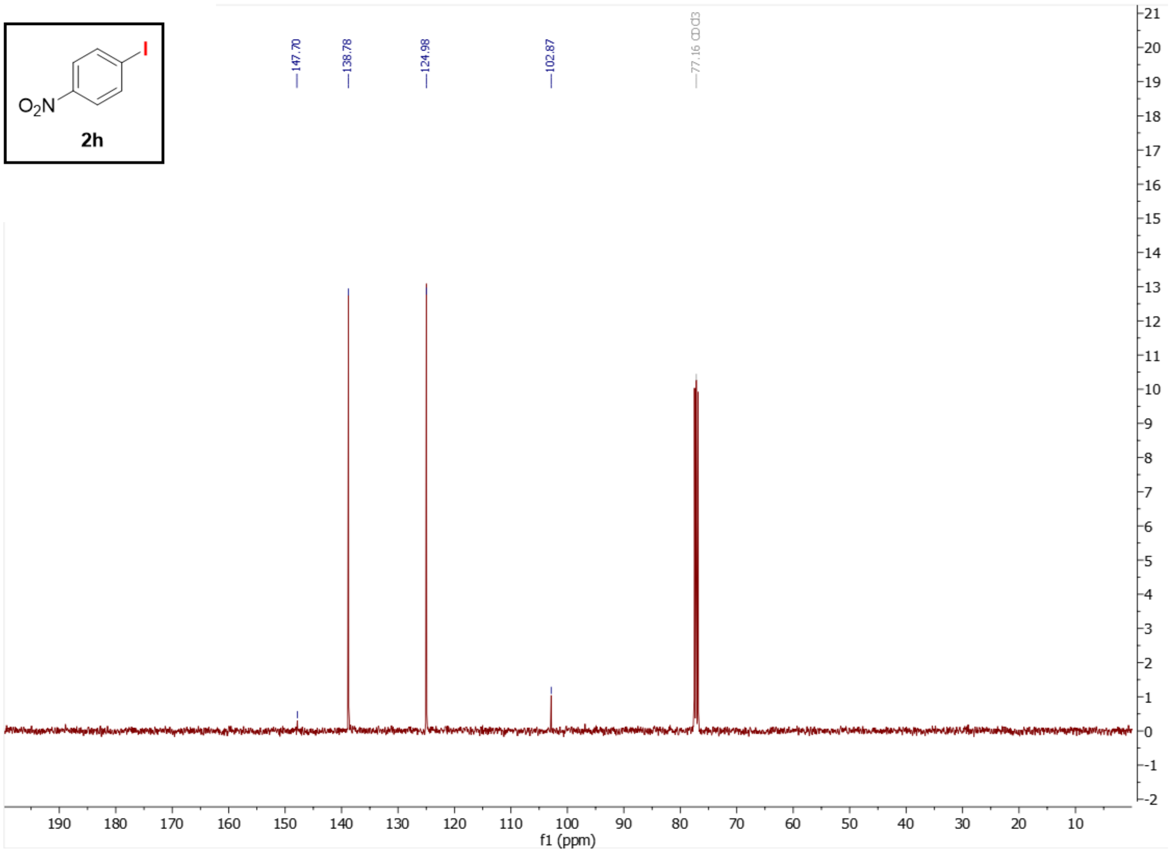

Figure S16. ^{13}C NMR (100 MHz, $CDCl_3$) of **2h**

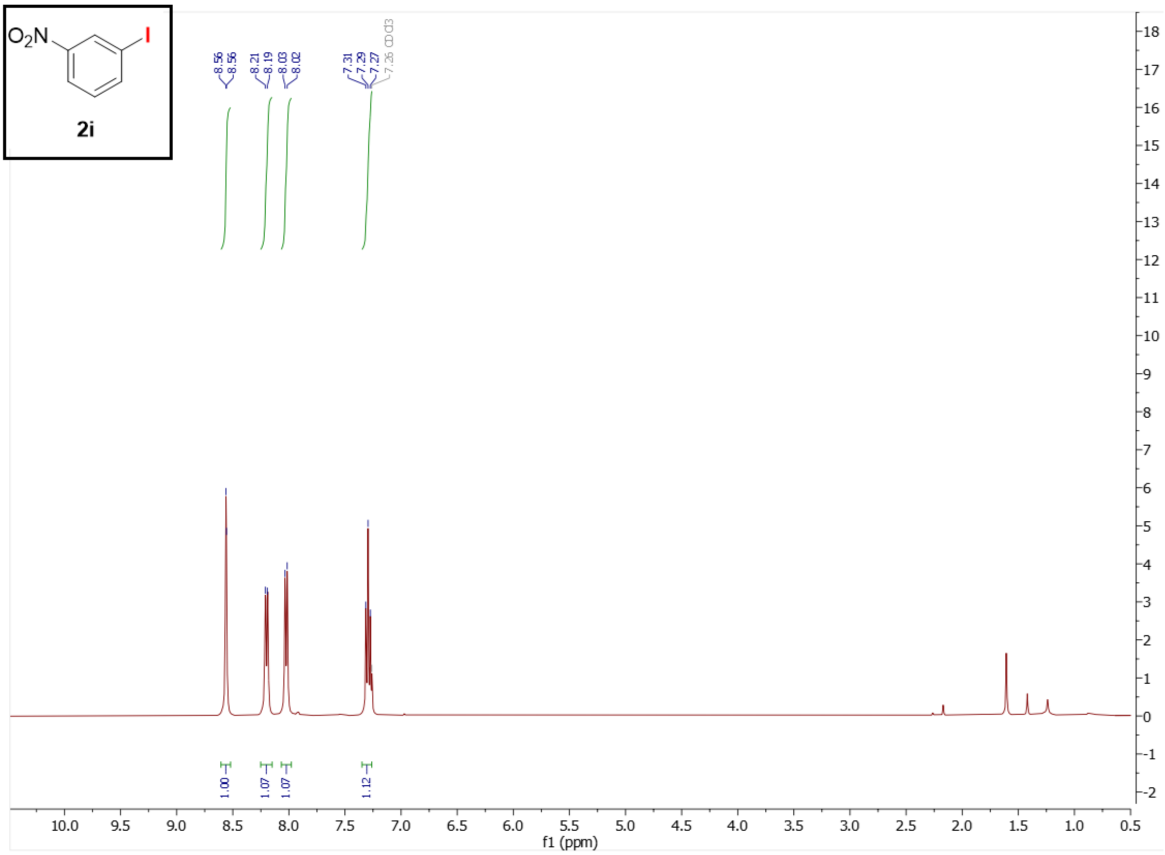

Figure S17. ^{1}H NMR (400 MHz, $CDCl_3$) of **2i**

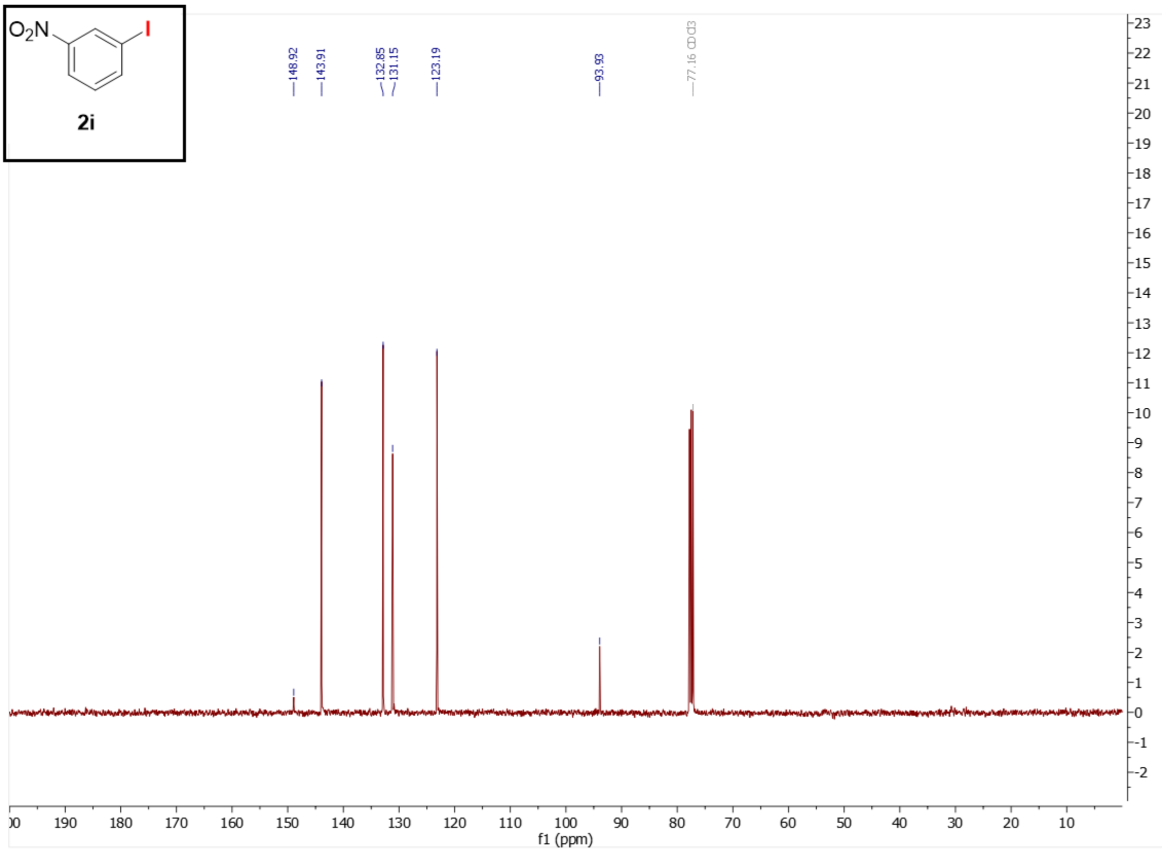

Figure S18. ^{13}C NMR (100 MHz, $CDCl_3$) of **2i**

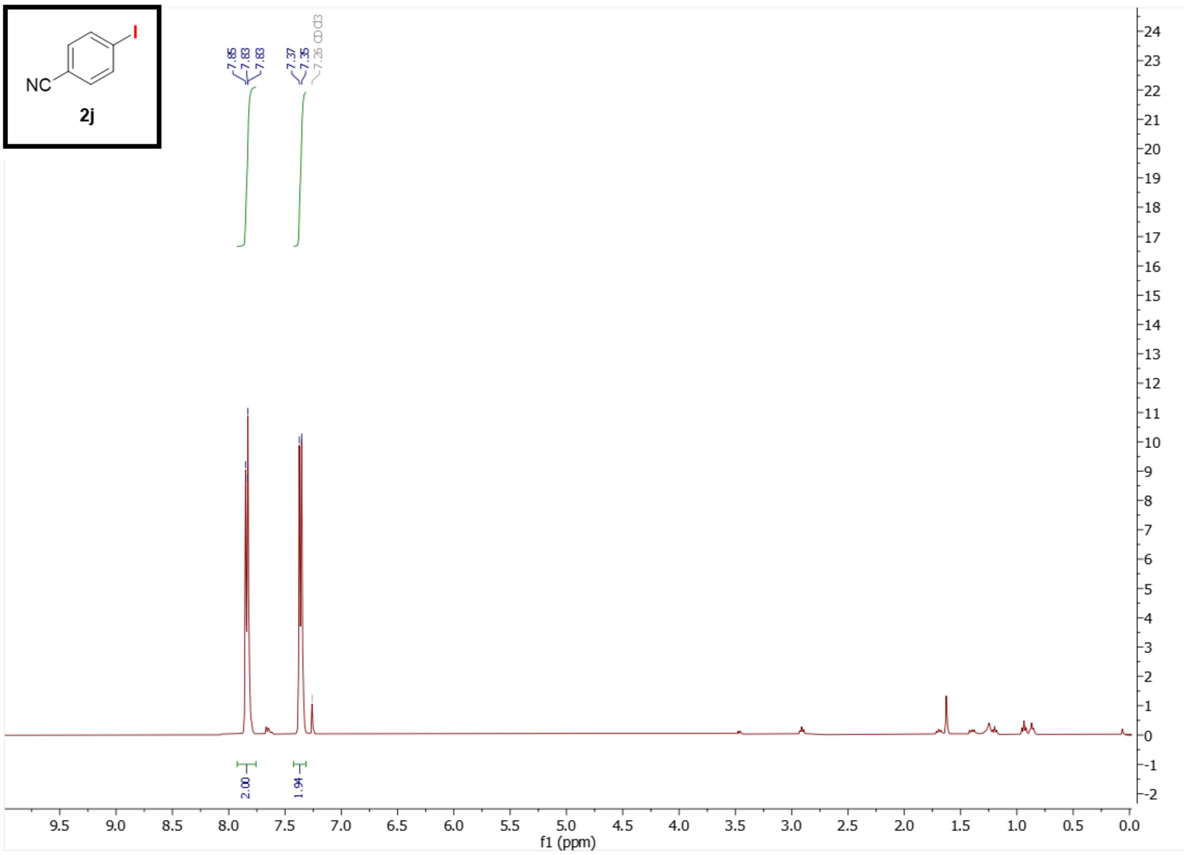

Figure S19. ^{1}H NMR (400 MHz, $CDCl_3$) of **2j**

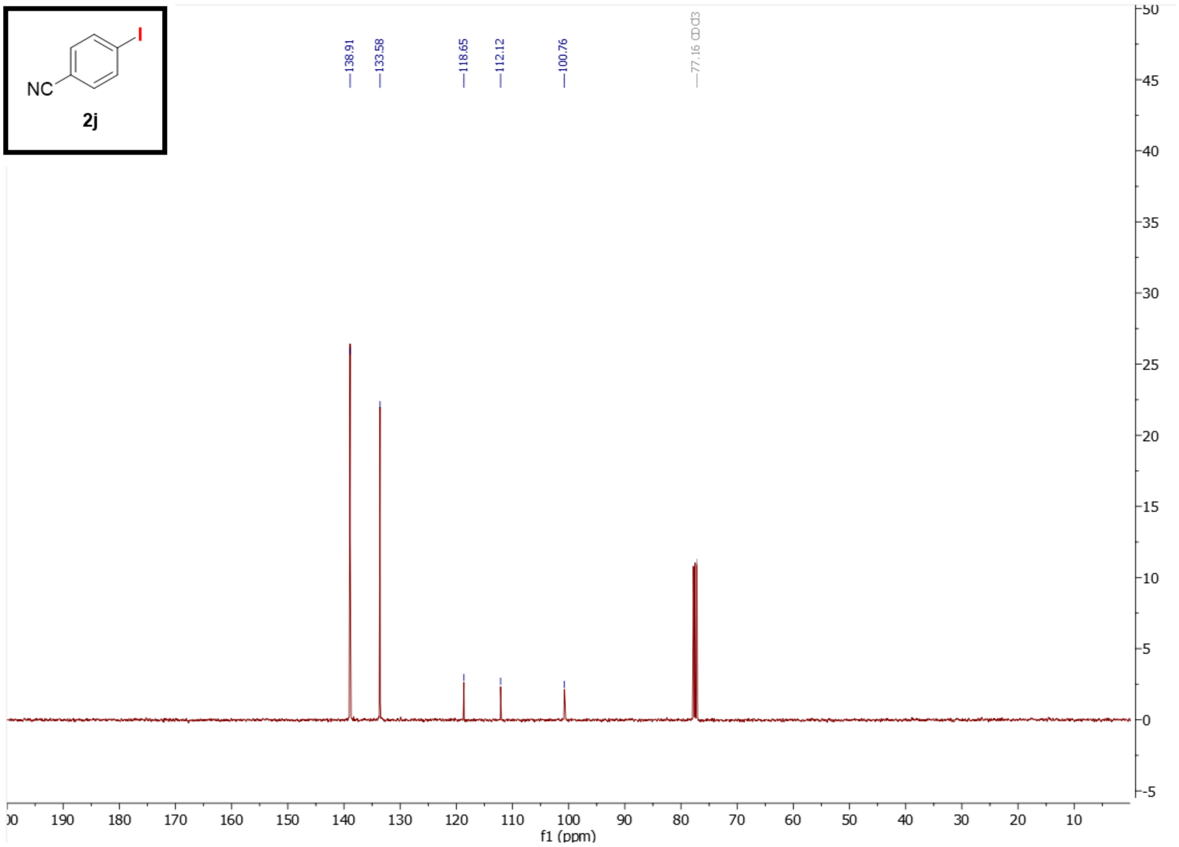

Figure S20. ^{13}C NMR (100 MHz, $CDCl_3$) of **2i**

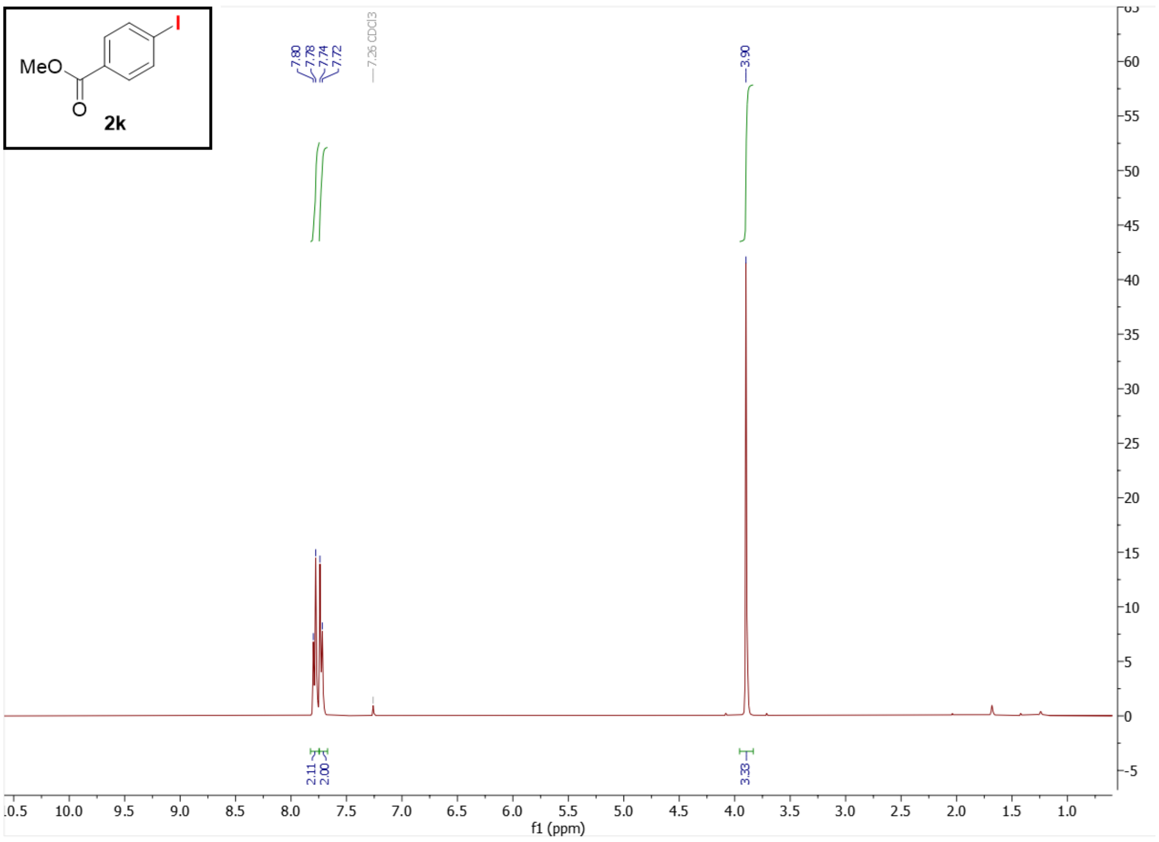

Figure S21. ^{1}H NMR (400 MHz, $CDCl_3$) of **2k**

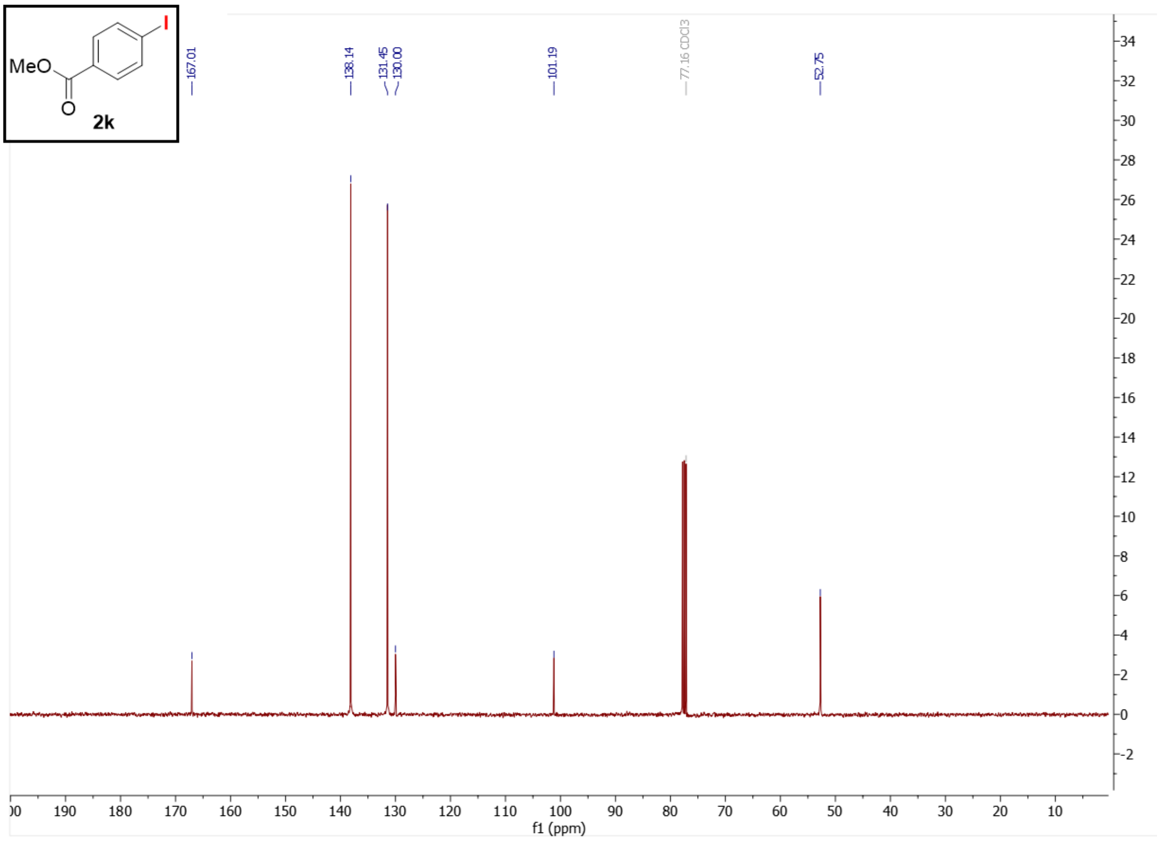

Figure S22. ^{13}C NMR (100 MHz, $CDCl_3$) of **2k**

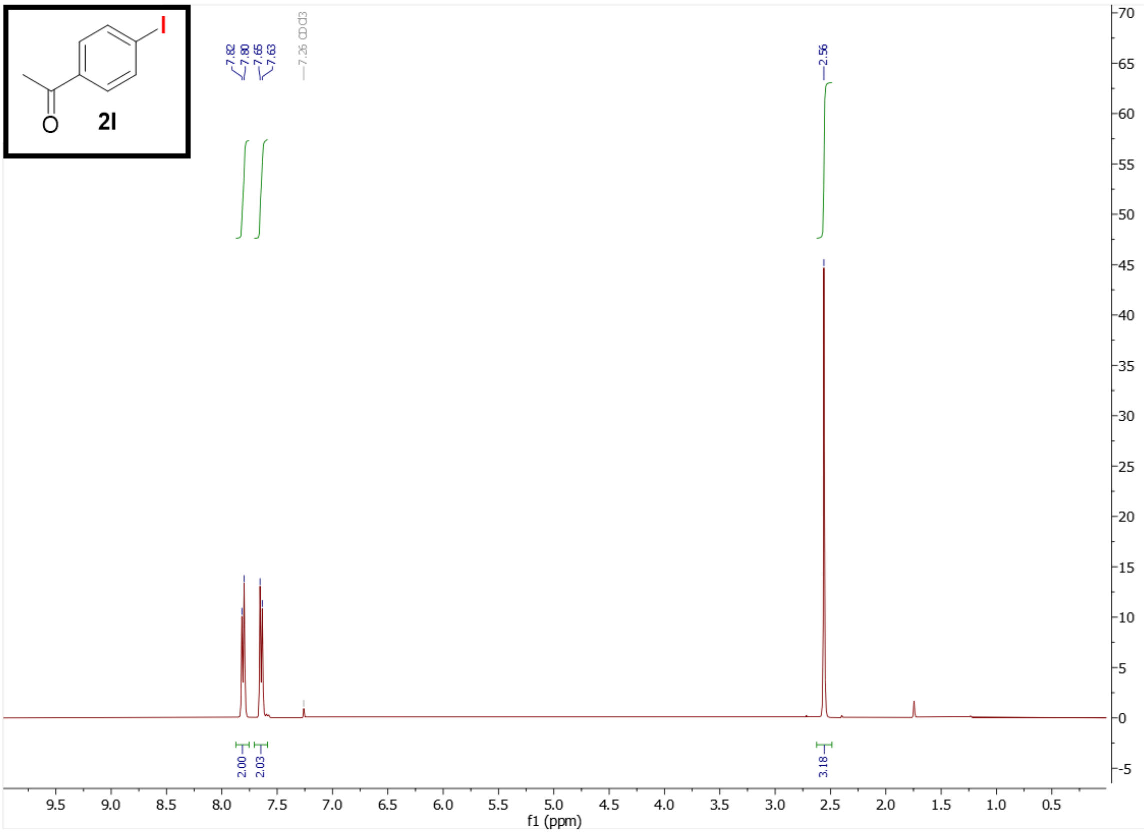

Figure S23. ^{1}H NMR (400 MHz, $CDCl_3$) of **2l**

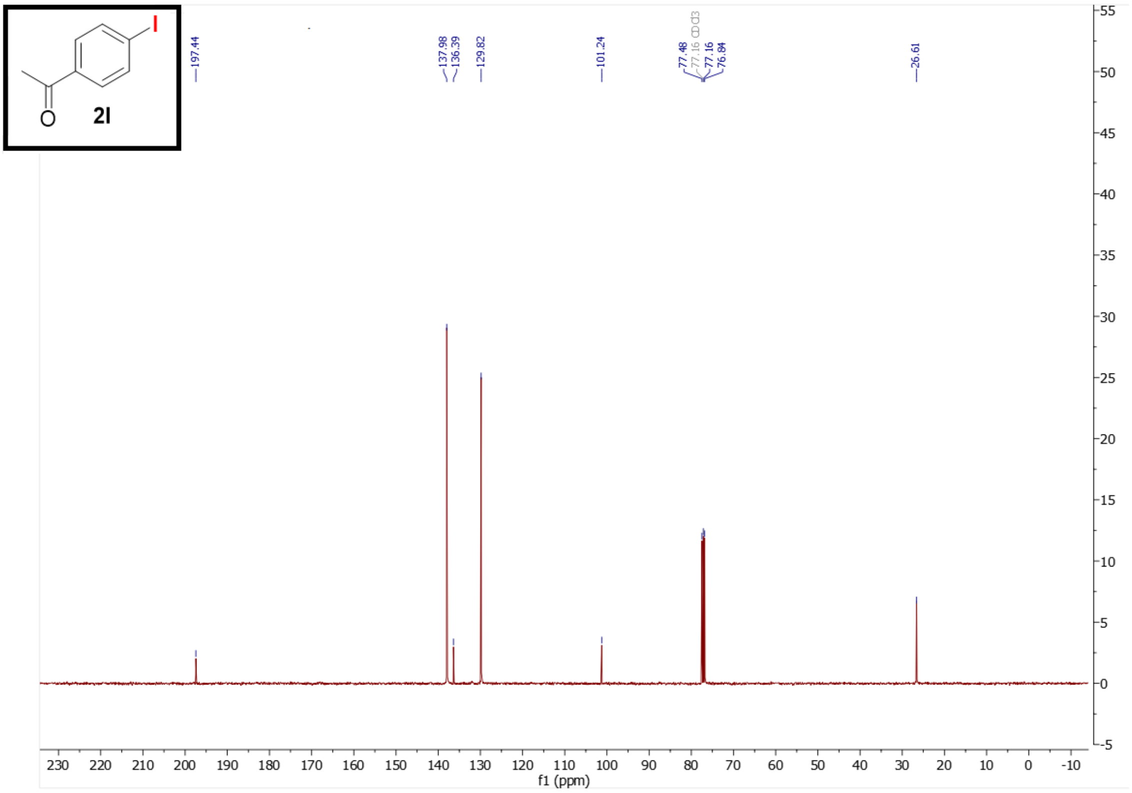

Figure S24. ^{13}C NMR (100 MHz, $CDCl_3$) of **2l**

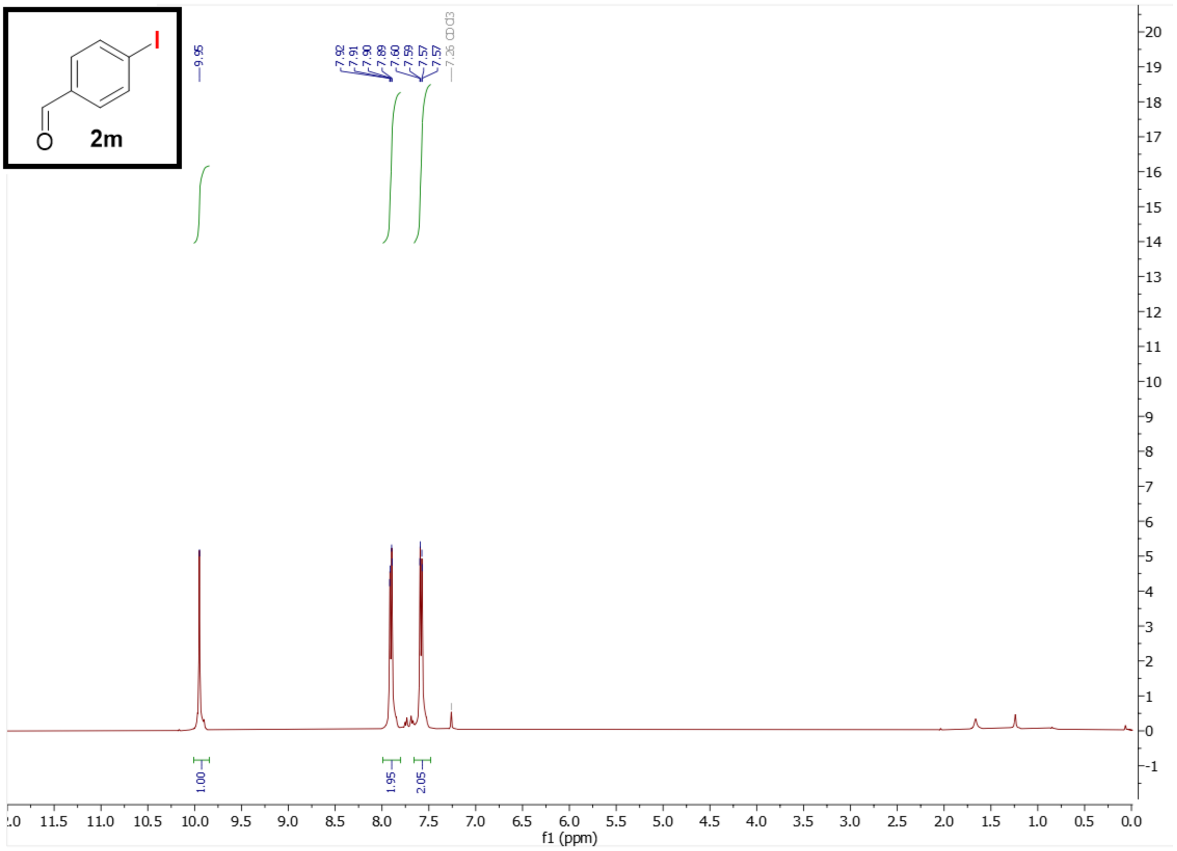

Figure S25. ^{1}H NMR (400 MHz, $CDCl_3$) of **2m**

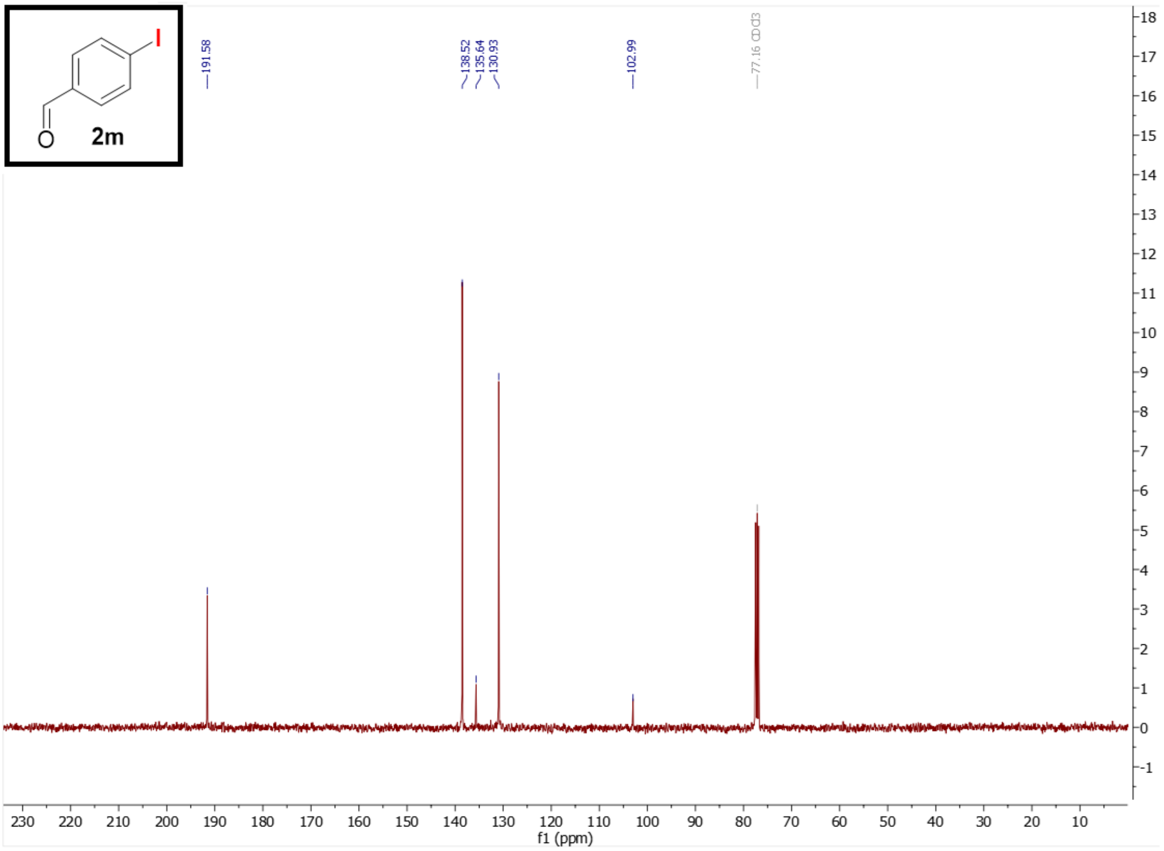

Figure S26. ^{13}C NMR (100 MHz, $CDCl_3$) of **2m**

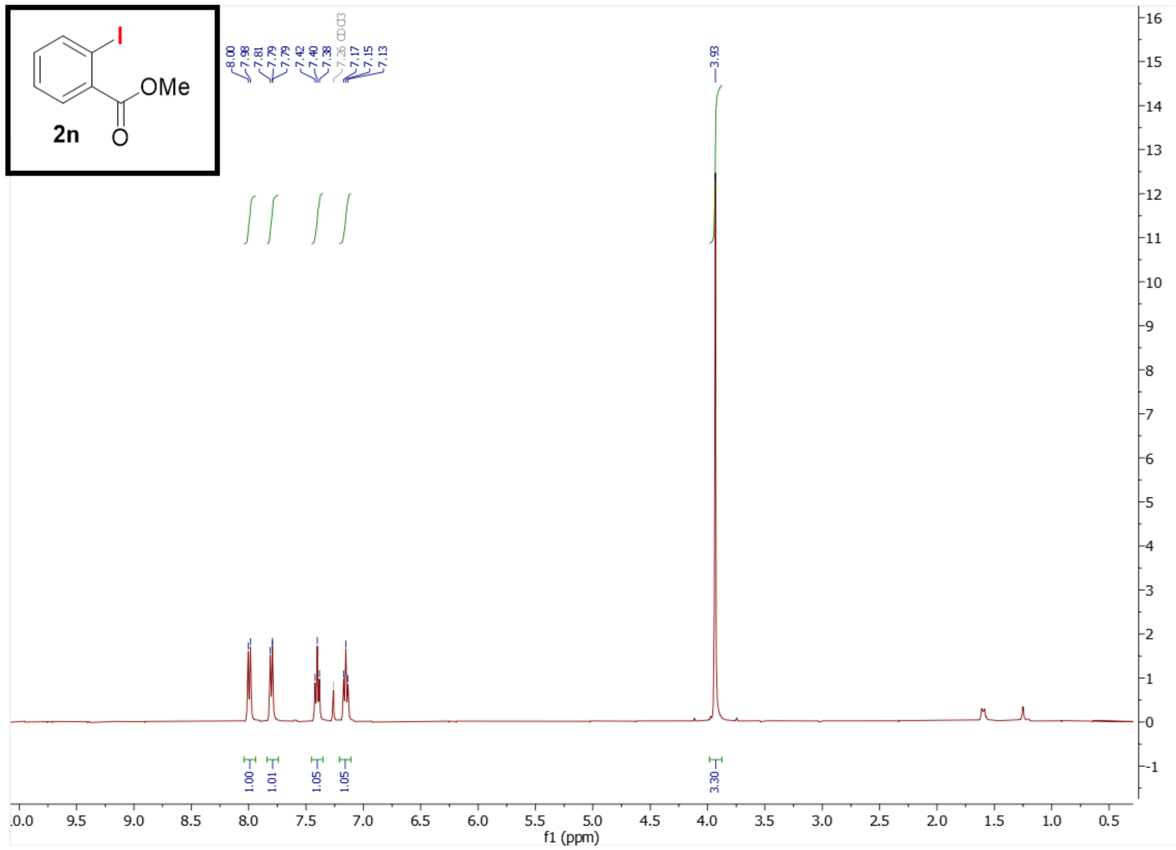

Figure S27. ^{1}H NMR (400 MHz, $CDCl_3$) of **2n**

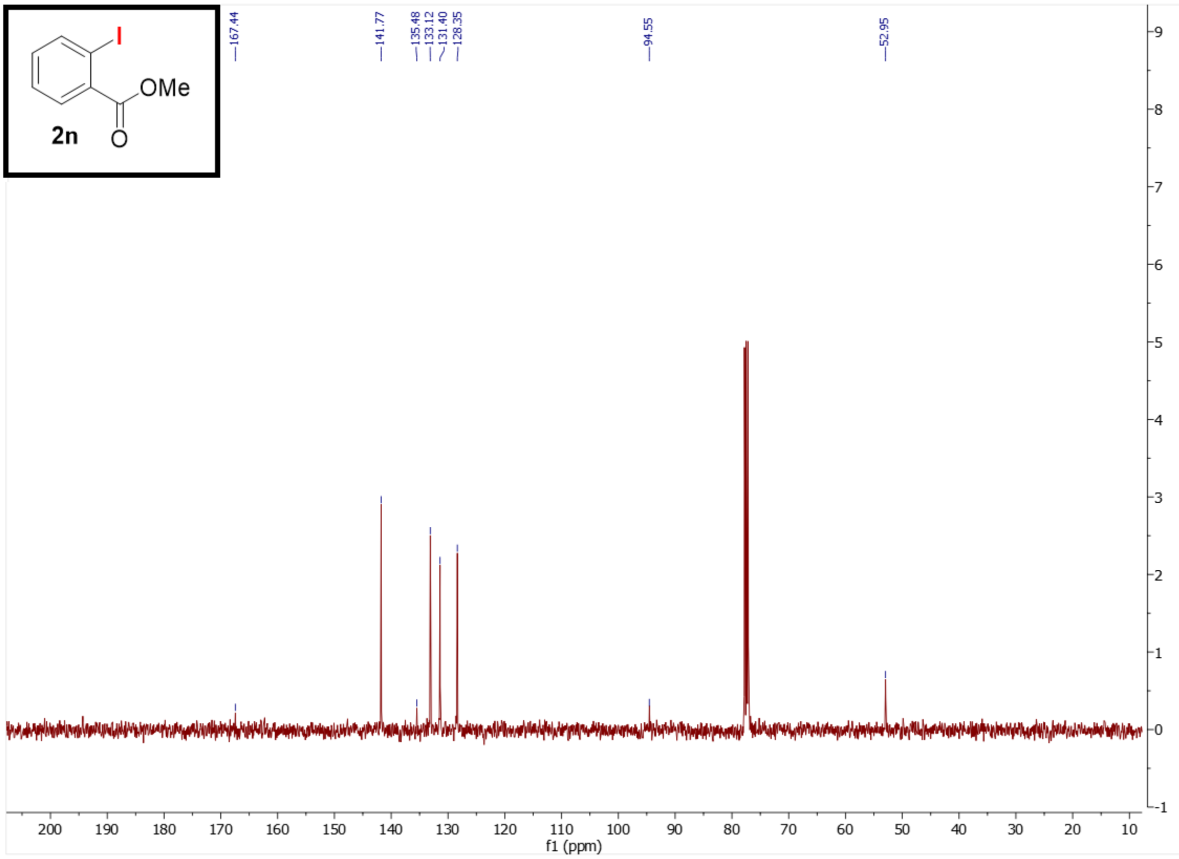

Figure S28. ^{13}C NMR (100 MHz, $CDCl_3$) of **2n**

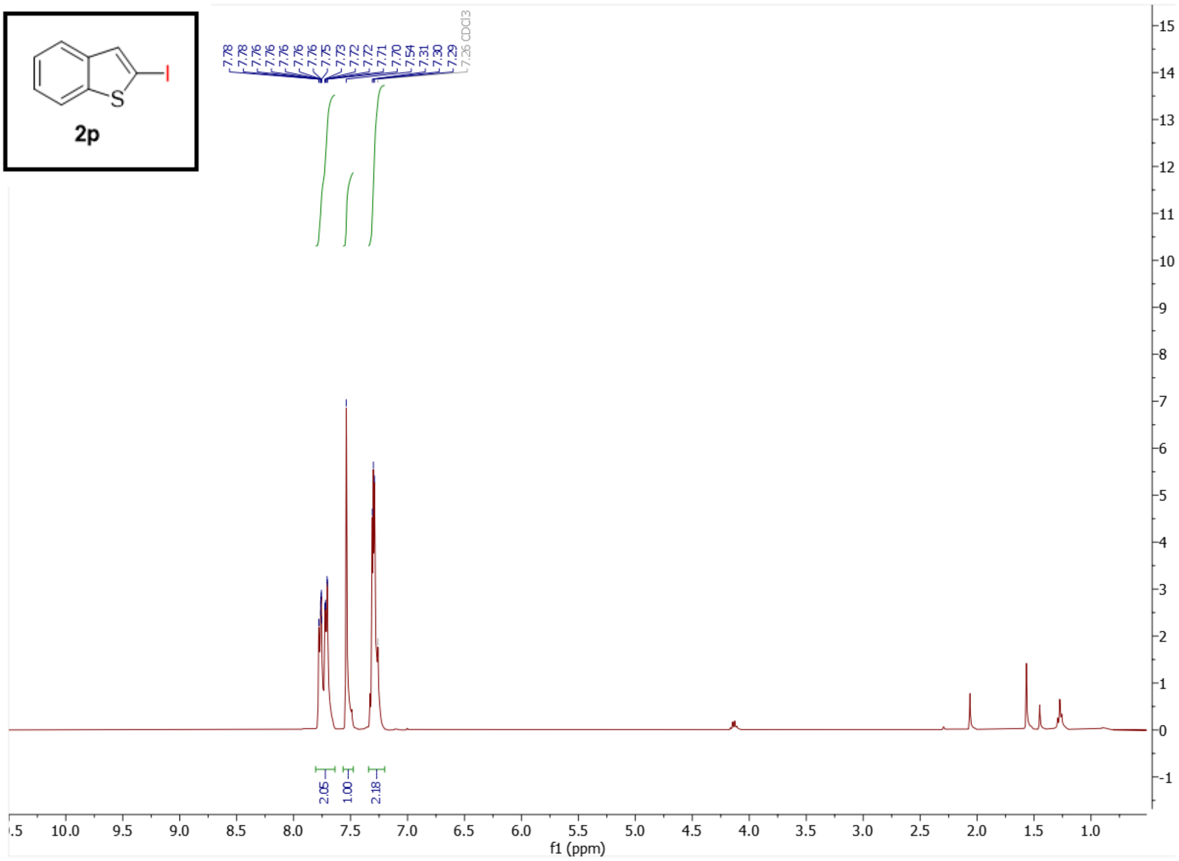

Figure S29. ^{1}H NMR (400 MHz, $CDCl_3$) of **2p**

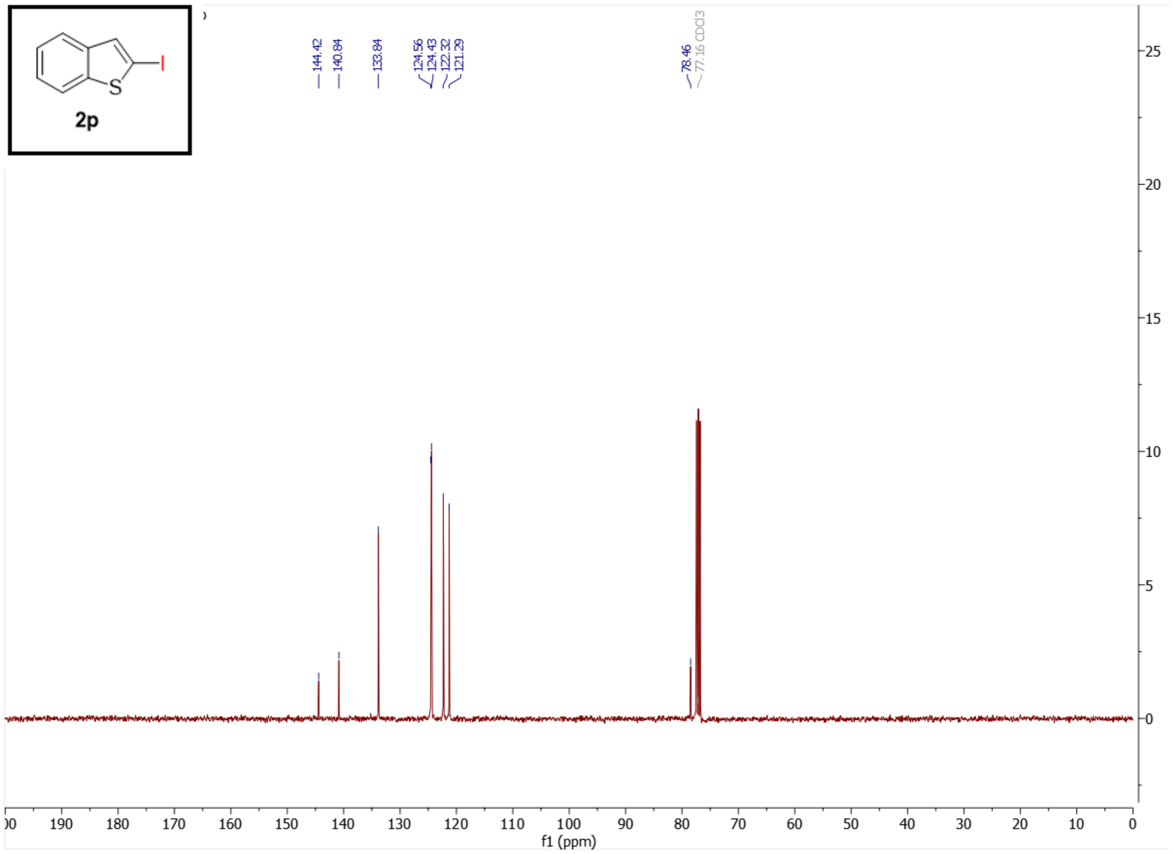

Figure S30. ^{13}C NMR (100 MHz, $CDCl_3$) of **2p**

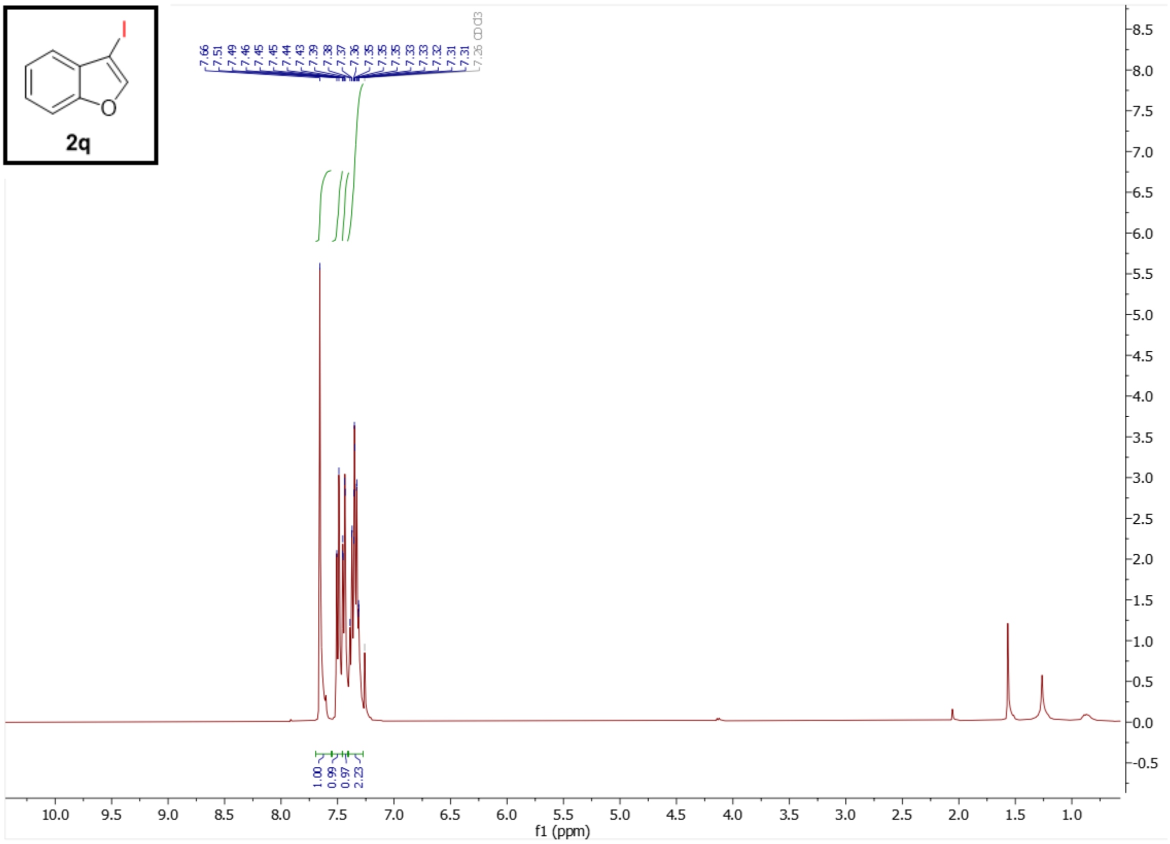

Figure S31. ^{1}H NMR (400 MHz, $CDCl_3$) of **2q**

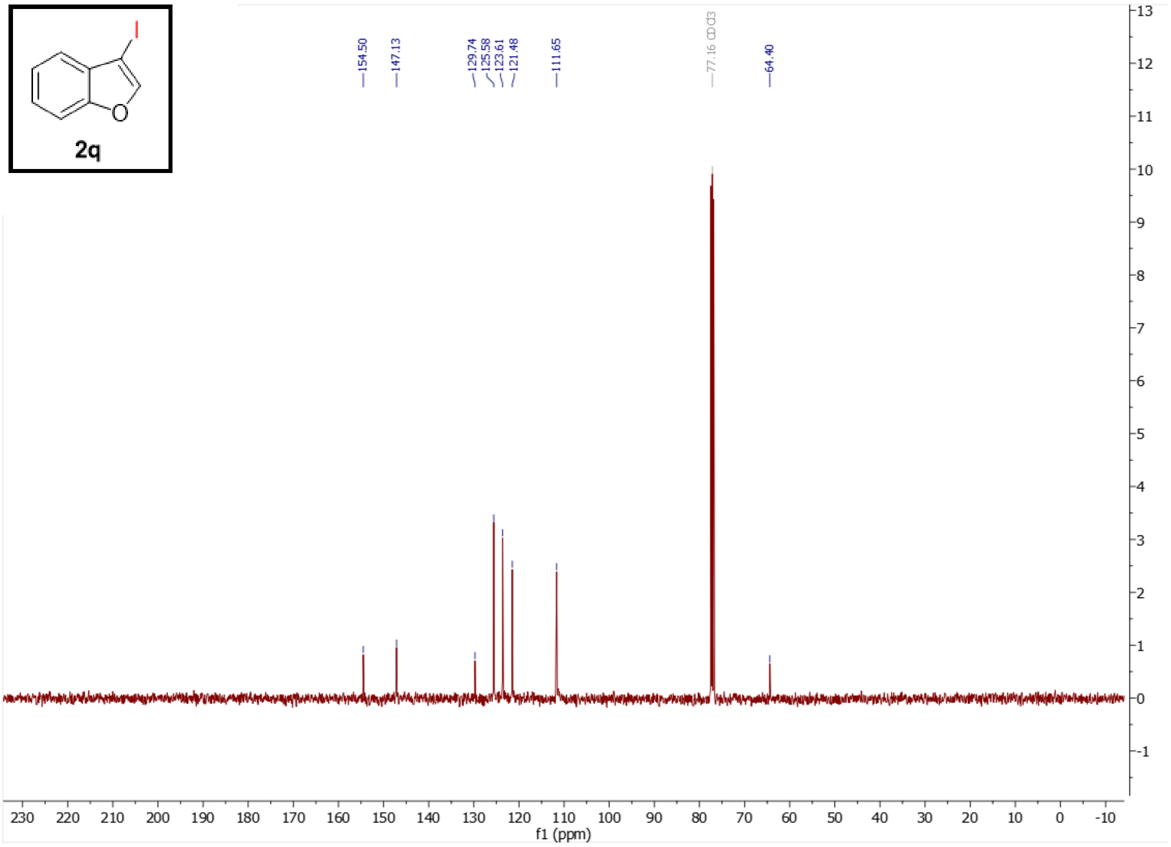

Figure S32. ^{13}C NMR (100 MHz, $CDCl_3$) of **2q**

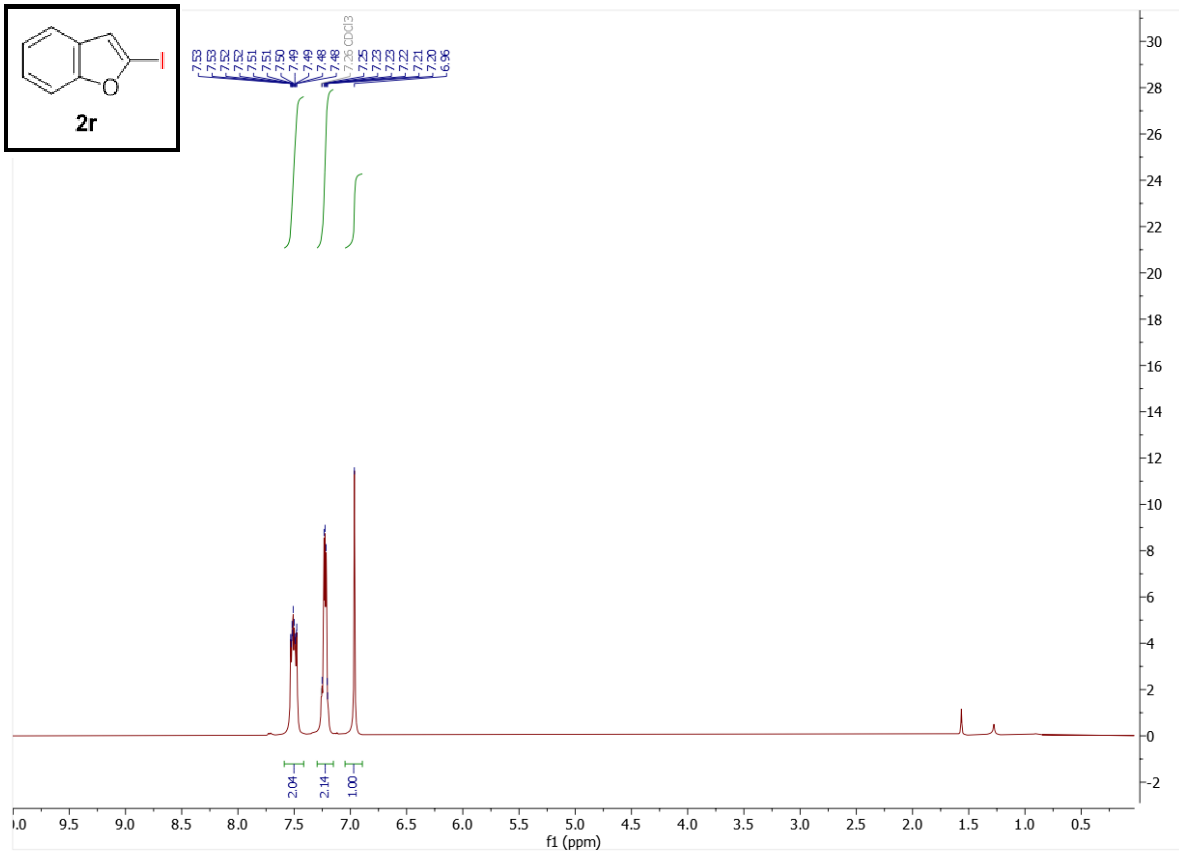

Figure S33. ^{1}H NMR (400 MHz, $CDCl_3$) of **2r**

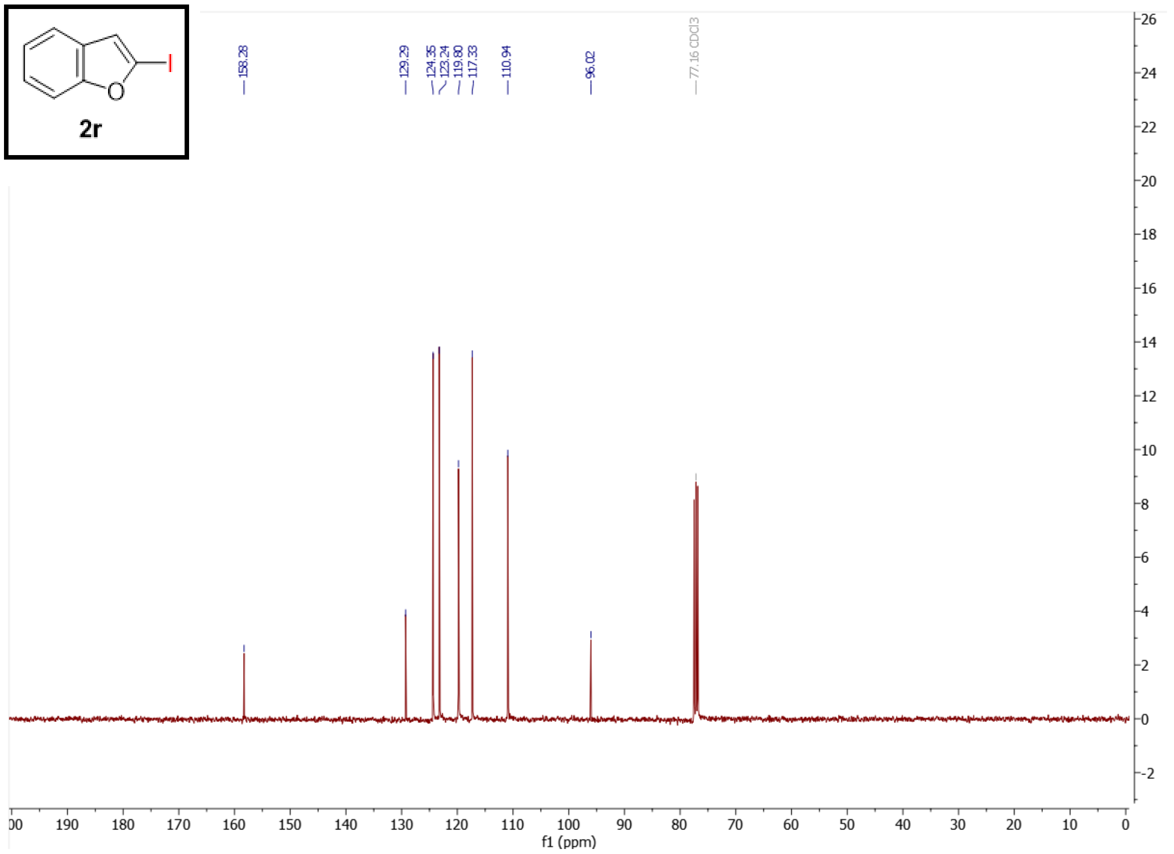

Figure S34. ^{13}C NMR (100 MHz, $CDCl_3$) of **2r**

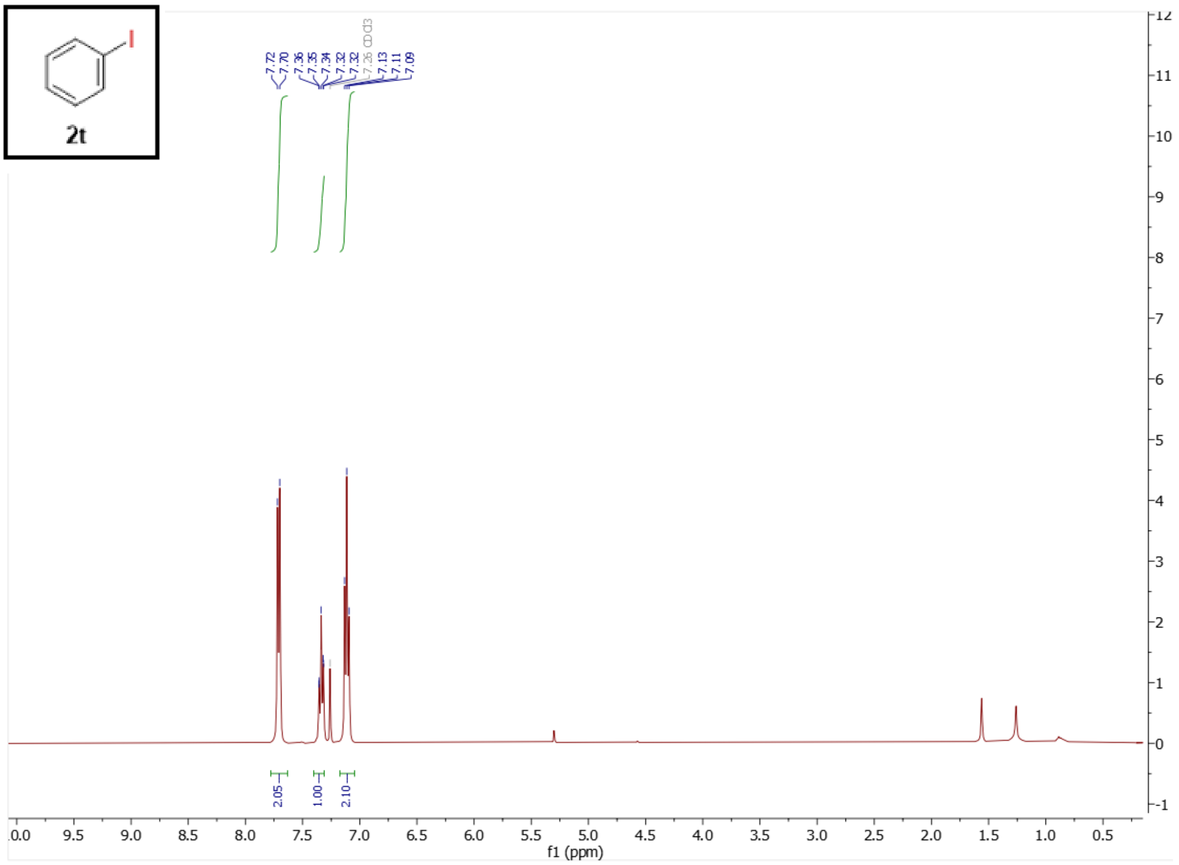

Figure S35. ^{1}H NMR (400 MHz, $CDCl_3$) of **2t**

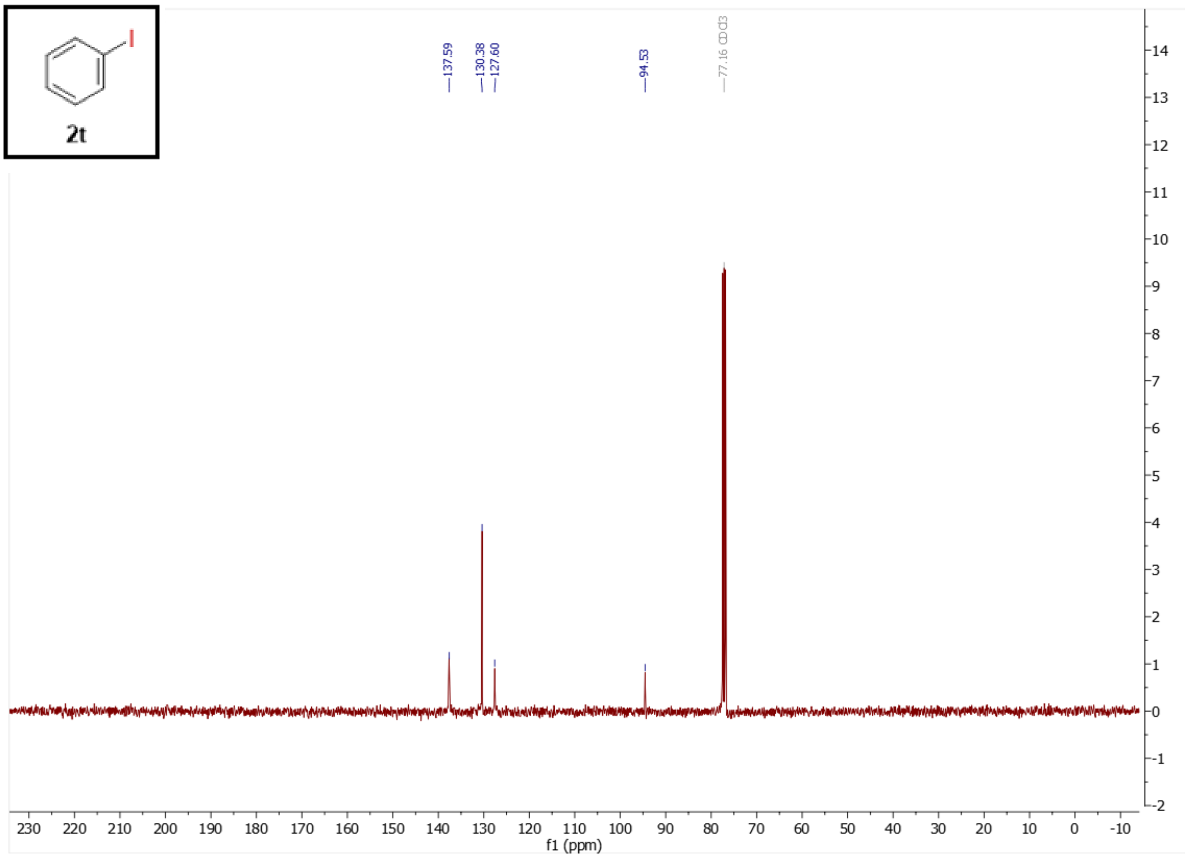

Figure S36. ^{13}C NMR (100 MHz, $CDCl_3$) of **2t**

3. Computational Details

Geometry optimization and energy calculations are performed using Gaussian 16 Rev. C.01 [1] employing the CAM-B3LYP density functional [2] and adopting the 6-31++g(d,p) basis set for all the elements, with the exception of I for which we employ the LANL2DZ basis sets. Free energies are evaluated taking into account thermal contributions, as implemented in Gaussian16. Solvation is taken into account by means of the Polarized Continuum Model (PCM) [3] as included in Gaussian 16. Stationary points are punctually verified by means of vibrational analysis calculations, i.e. checking that (i) no imaginary frequency appears for energy minima (reactants and products) and (i) exactly one imaginary frequency is calculated for saddle points (transition states) on the potential energy surface.

4. Charge distribution of 1a and 1a’

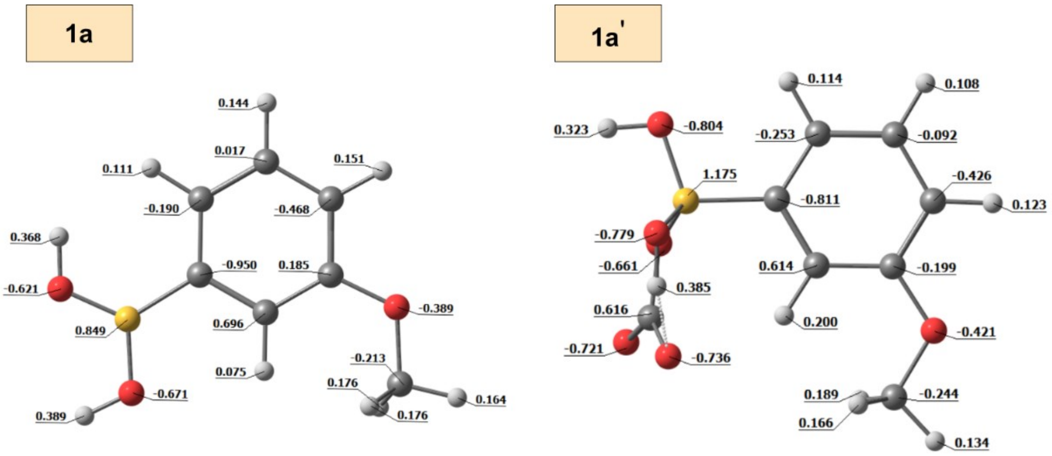

Calculated Hirshfeld charges of the reference *m*-methoxyphenylboronic acid (**1a**) and its corresponding intermediate (**1a’**, cf. *main text*), showing the nucleophilic character associated to the *ipso* carbon, and thus the reaction selectivity experimentally observed.

5. Thermochemical data

**solvent**	ΔG_{coord}	$\Delta G^{\ddagger}$	$\Delta G_{reaction}$
DMC	-22.47	13.24	-109.95
THF	-10.85	16.24	-86.77
MeTHF	-11.38	15.93	-87.86
H_2O	-3.12	18.47	-69.72
ACN	-6.44	17.96	-73.72

Energy values (in kcal/mol units) for reagents, the free energy variation accompanying the reaction of tetracoordination (ΔG_{coord}), the activation energy ($\Delta G^{\ddagger}$, i.e. the energy difference

between **2a’** and **1a’**) and the free energy variation ($\Delta G_{reaction}$) for the *ipso* iodination reaction of **1a** evaluated at the CAM-B3LYP/6-31++G(d,p) – LANL2DZ level of theory

6. Energy profiles for the KF-mediated *ipso*-iodination

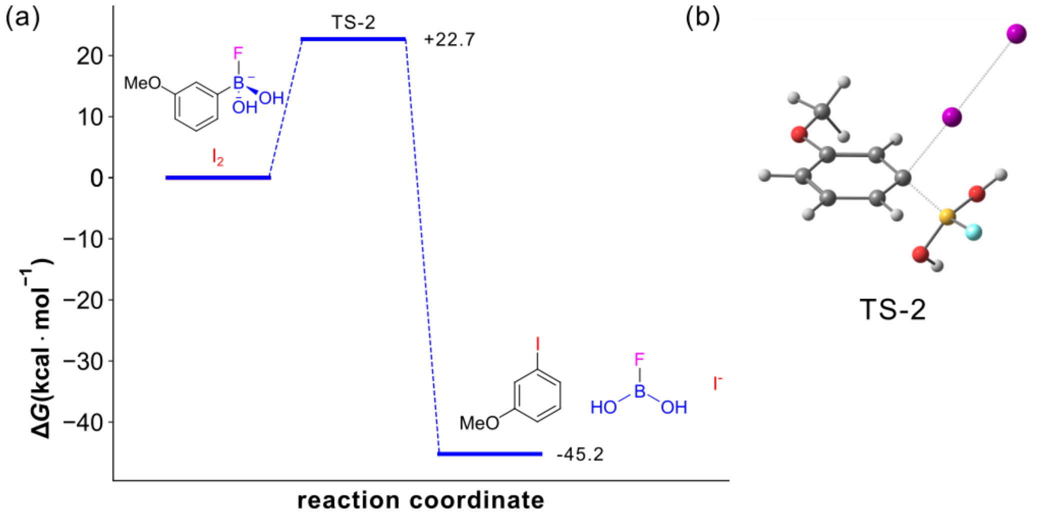

a) Free energy profile for the iodination reaction of **1a** in DMC, using KF as a Lewis base. Free energy is referred to that of the tetracoordinated intermediate. b) Optimized structure for the transition state TS-2.

7. Energy profiles for the ipso-iodination of phenyl trifluoroboronate without base

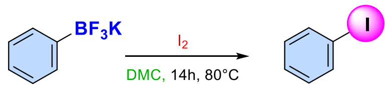

1t' **2t** (26%)

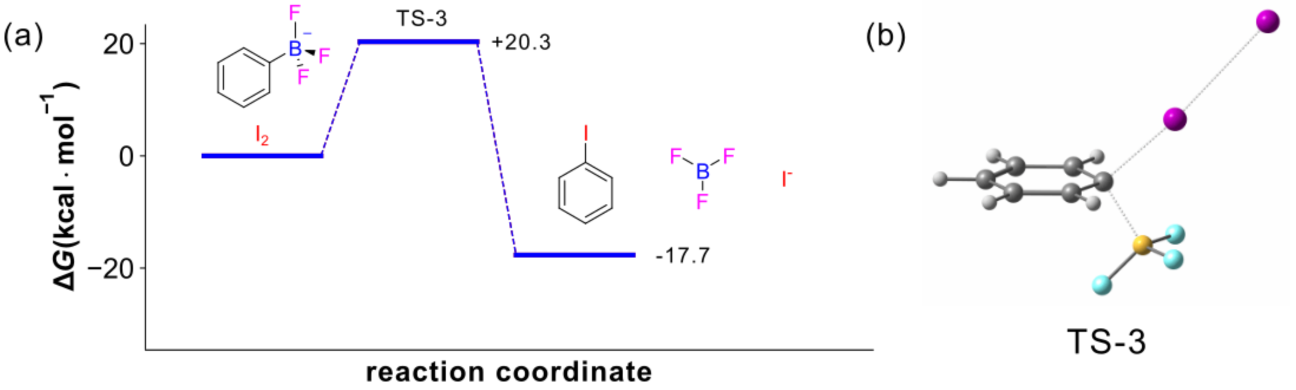

a) Free energy profile for the iodination reaction of phenyl trifluoroboronate in DMC, conducted without any Lewis base. Free energy is referred to the sum of free energies of the reagents. b) Optimized structure for the transition state TS-3.

8. Optimized structures

$BC_8H_9O_6I_2$ (2a')

6	1.195750000	0.892063000	1.990916000
6	2.184677000	2.264149000	-0.220205000
6	1.282508000	2.288599000	2.020090000
6	1.623303000	0.147982000	0.861623000
6	2.067444000	0.891513000	-0.266193000
6	1.781531000	2.972868000	0.925434000
5	2.179932000	-1.446113000	1.030858000
8	2.699618000	3.026280000	-1.240871000
6	3.213605000	2.317325000	-2.357827000
8	3.506923000	-1.337904000	1.617123000
8	1.264110000	-2.112702000	1.950223000
8	2.196327000	-2.117442000	-0.289801000
6	3.219652000	-2.057293000	-1.203348000
8	3.108781000	-2.784059000	-2.211102000
8	4.180394000	-1.249009000	-0.954447000
1	0.867175000	0.334419000	2.862035000
1	0.954848000	2.840828000	2.897651000
1	2.403410000	0.323120000	-1.122060000
1	1.870806000	4.055771000	0.923997000
1	2.412234000	1.810941000	-2.908406000
1	3.951858000	1.567156000	-2.054081000
1	3.678007000	3.068605000	-3.000596000
1	4.091662000	-1.222331000	0.836806000
1	1.722988000	-2.903883000	2.249456000
53	-1.034216000	-0.013669000	0.344011000
53	-4.020741000	-0.143852000	-0.481955000

$BC_8H_9O_6$ (1a')

6	-1.288060000	-1.999096000	-0.321548000
6	-2.225236000	0.587660000	-0.011758000
6	-2.647167000	-1.721106000	-0.485560000
6	-0.368625000	-0.995040000	0.007912000
6	-0.875446000	0.308567000	0.160797000
6	-3.132544000	-0.424664000	-0.332537000
5	1.244553000	-1.272669000	0.250933000
8	-2.765076000	1.861349000	0.116088000
6	-1.856140000	2.903696000	0.398447000
8	1.632149000	-1.017774000	1.640289000
8	1.501252000	-2.680212000	-0.106819000
8	2.059048000	-0.414828000	-0.691741000
6	2.440581000	0.875797000	-0.474474000
8	3.141653000	1.410256000	-1.367809000
8	2.057575000	1.439582000	0.608891000
1	-0.915041000	-3.011638000	-0.451423000

1	-3.342691000	-2.521616000	-0.740547000
1	-0.155149000	1.082927000	0.410749000
1	-4.184274000	-0.180858000	-0.460836000
1	-1.080494000	2.988030000	-0.370911000
1	-1.354688000	2.755948000	1.362278000
1	-2.449018000	3.822689000	0.429766000
1	1.822212000	-0.057493000	1.646727000
1	2.453375000	-2.786362000	-0.012648000

C_7H_7OI (2a)

6	0.000000000	0.481328000	0.000000000
6	-2.665066000	1.233579000	0.000000000
6	-0.317494000	1.830164000	0.000000000
6	-0.981562000	-0.508238000	0.000000000
6	-2.322979000	-0.122424000	0.000000000
6	-1.667054000	2.193127000	0.000000000
53	2.038987000	-0.110540000	0.000000000
8	-3.361041000	-0.998453000	0.000000000
6	-3.079191000	-2.389436000	0.000000000
1	-3.714243000	1.507459000	0.000000000
1	0.456223000	2.588053000	0.000000000
1	-0.700545000	-1.552181000	0.000000000
1	-1.935040000	3.244920000	0.000000000
1	-2.519102000	-2.679958000	0.895302000
1	-4.046091000	-2.890722000	0.000000000
1	-2.519102000	-2.679958000	-0.895302000

$B(OH)_2CO_3^-$

5	-1.161439000	-0.031425000	0.000201000
8	-2.318429000	-0.777917000	-0.000546000
8	-1.241281000	1.319326000	0.000539000
8	0.037102000	-0.725570000	0.000566000
6	1.312149000	-0.094875000	0.000108000
8	1.302148000	1.165105000	-0.000893000
8	2.263051000	-0.876532000	0.000142000
1	-2.119325000	-1.721082000	-0.001136000
1	-0.287097000	1.612167000	0.001023000

$BC_7H_9O_3I_2F$ (TS2)

6	1.760480683	0.500955458	0.284115780
6	3.551848861	-1.256012308	1.545241087
6	2.354340268	-0.579413744	-0.428710258
6	2.066210087	0.627128820	1.659830906
6	2.968597227	-0.230048584	2.276440339
6	3.248040545	-1.434863666	0.186225005
5	1.884879375	2.060430223	-0.596489013
8	3.881398226	-2.477649494	-0.422554131

6	3.595147667	-2.718263468	-1.791376777
8	3.288957355	2.348053542	-0.559036181
8	1.413534346	1.952771456	-1.941945680
9	1.119071682	2.978612605	0.176911445
1	4.253864390	-1.942822267	2.008040067
1	2.110647773	-0.679650551	-1.479520828
1	1.604579811	1.433170246	2.220547437
1	3.215892355	-0.110789573	3.326359720
1	4.190165568	-3.585947884	-2.074447800
1	3.880152638	-1.861920697	-2.411950033
1	2.532795399	-2.939654735	-1.939988857
1	3.534494096	2.798648440	-1.374198887
1	0.452520228	1.996931117	-1.984013059
53	-0.614497243	0.132160334	0.177835152
53	-3.747960600	-0.438951423	-0.062795420

$BC_6H_5I_2F_3$ (TS3)

1	-2.107616149	-0.343487156	-2.151584248
6	-2.487803162	-0.733764196	-1.212710183
6	-3.454388197	-1.722010302	1.215957993
6	-2.001823149	-0.173231140	-0.000082095
6	-3.454489201	-1.722091303	-1.215868181
6	-3.935768215	-2.212954357	0.000080905
6	-2.487799162	-0.733597198	1.212640993
1	-4.692572243	-2.992022438	0.000139905
1	-2.107786152	-0.342991156	2.151447059
1	-3.833187210	-2.118595344	2.151946062
5	-2.464624246	1.594929973	-0.000007095
9	-1.961595227	2.159317030	1.153792985
9	-1.961733231	2.159421030	-1.153807178
9	-3.854251343	1.593499919	0.000041905
1	-3.833395214	-2.118727347	-2.151792252
53	0.302477012	-0.033267047	-0.000019095
53	3.581076252	-0.145658939	0.000009905

$B(OH)_2F$

5	0.000000000	0.018131000	0.000000000
8	-0.560483000	-1.218235000	0.000000000
8	-0.741873000	1.148277000	0.000000000
9	1.337521000	0.158300000	0.000000000
1	0.074075000	-1.944476000	0.000000000
1	-1.692913000	0.988790000	0.000000000

9. References.

[1] Gaussian 16, Revision C.01, M. J. Frisch, G. W. Trucks, H. B. Schlegel, G. E. Scuseria, M. A. Robb, J. R. Cheeseman, G. Scalmani, V. Barone, G. A. Petersson, H. Nakatsuji, X. Li, M. Caricato, A. V. Marenich, J. Bloino, B. G. Janesko, R. Gomperts, B. Mennucci, H. P. Hratchian, J. V. Ortiz, A. F. Izmaylov, J. L. Sonnenberg, D. Williams-Young, F. Ding, F. Lipparini, F. Egidi, J. Goings, B. Peng, A. Petrone, T. Henderson, D. Ranasinghe, V. G. Zakrzewski, J. Gao, N. Rega, G. Zheng, W. Liang, M. Hada, M. Ehara, K. Toyota, R. Fukuda, J. Hasegawa, M. Ishida, T. Nakajima, Y. Honda, O. Kitao, H. Nakai, T. Vreven, K. Throssell, J. A. Montgomery Jr., J. E. Peralta, F. Ogliaro, M. J. Bearpark, J. J. Heyd, E. N. Brothers, K. N. Kudin, V. N. Staroverov, T. A. Keith, R. Kobayashi, J. Normand, K. Raghavachari, A. P. Rendell, J. C. Burant, S. S. Iyengar, J. Tomasi, M. Cossi, J. M. Millam, M. Klene, C. Adamo, R. Cammi, J. W. Ochterski, R. L. Martin, K. Morokuma, O. Farkas, J. B. Foresman, D. J. Fox, Gaussian, Inc., Wallingford CT, 2016.

[2] Takeshi Yanai, David P Tew, Nicholas C Handy, A new hybrid exchange–correlation functional using the Coulomb-attenuating method (CAM-B3LYP), *Chemical Physics Letters*, **2004,** *393*, 51-57. ISSN 0009-2614, https://doi.org/10.1016/j.cplett.2004.06.011.

[3] J. Tomasi, B. Mennucci, and R. Cammi, Quantum mechanical continuum solvation models, *Chem. Rev.*, **2005**, *105*, 2999-3093. DOI: 10.1021/cr9904009.
